# Riboflavin inhibits growth and reduces virulence of *Cryptococcus neoformans in vitro* by membrane disruption and excessive accumulation of reactive oxygen species and exhibits efficacy against pulmonary cryptococcosis and meningitis

**DOI:** 10.1080/21505594.2025.2543064

**Published:** 2025-08-03

**Authors:** Jian Huang, Anni Ge, Junwen Lei, Quan Zhou, Shu Gong, Caiyan Xin, Zhangyong Song

**Affiliations:** a School of Basic Medical Sciences, Southwest Medical University, Luzhou, People’s Republic of China; b Department of Medical Technology, Xichang Medical College, Xichang, People’s Republic of China; c The Public Platform of Molecular Biotechnology, Public Center of Experimental Technology, Southwest Medical University, Luzhou, People’s Republic of China; d Hemodynamics and Medical Engineering Combination Key Laboratory of Luzhou, Southwest Medical University, Luzhou, Sichuan, China

**Keywords:** *Cryptococcus neoformans*, *cryptococcus* infection, riboflavin, cell membrane, virulence

## Abstract

The incidences of pulmonary cryptococcosis and meningitis cause significant morbidity and mortality. Effective and affordable drugs for treatment of cryptococcal meningitis are urgently needed. Drug reuse is an effective strategy for the development of new antifungals against *Cryptococcus neoformans* infection. In this study, riboflavin (RF) significantly inhibited growth of *C. neoformans* as determined by the broth microdilution and spot dilution methods. Moreover, RF significantly inhibited biofilm formation and reduced virulence (capsule, melanin, and urease). In addition, RF caused cell membrane damage, compromised cell wall integrity, and promoted accumulation of intracellular reactive oxygen species (ROS). RT-qPCR analysis confirmed that RF treatment up-regulated expression genes related to cell wall biosynthesis (*CHS3*, *CDA1*, and *FKS1*), the cell wall damage repair pathway (*Pkc1* and *Mpk1*), and virulence (*CAP59*, *Lac1*, *Lac2*), however, *Ure1* were down-regulated after RF treatment. Finally, in mouse models of intranasal and intravenous infection, RF treatment significantly reduced the fungal burden in multiple organs, reduced lung and brain damage, and decreased the levels of plasma interferon (IFN)-γ, tumor necrosis factor (TNF)-α, and interleukin (IL)-4 in the early stage of infection. These results showed that RF exerted significant antifungal effects for treatment of *C. neoformans* infection.

## Introduction

*Cryptococcus neoformans* is an environmental basidiomycete yeast found in bird droppings. Human cryptococcal infection is usually caused by inhalation of basidiospores or dried yeast cells, often accompanied by pneumonia of unknown etiology, resulting in the formation of granulomas containing viable yeast cells and production of antibodies via the adaptive immune response [1,2], followed goes into dormancy [2,3]. Eventually, *C*. *neoformans* is reactivated in individuals with immunosuppression and spreads in the blood to various organs, leading to the most dangerous form, cryptococcal meningitis (CM) [4]. An estimated 152,000 cases of CM are reported annually worldwide, resulting in 112,000 cryptococcal-related deaths. Globally, cryptococcal infection accounts for 147,000 (19%) deaths associated with acquired immunodeficiency syndrome [5,6]. Moreover, in low-income areas of Africa, the mortality rate of HIV-associated CM is about 70% [7].

Cryptococcosis is usually fatal unless treated with antifungal drugs. However, current treatment options are hampered by toxicity and pathogen resistance [8]. Standard care for CM includes a short course of intravenous annotated liposomal amphotericin B combined with oral fluorocytosine, followed by fluconazole monotherapy as a consolidation therapy [9]. Up to 60% of meningitis patients relapse after treatment due to clinical resistance to fluconazole [10,11]. Meanwhile, inadequate medical care and the expense of standard antimicrobials have resulted in increased use of fluconazole monotherapy [12]. Therefore, the development of new antifungal drugs must be accelerated to slow the spread of CM. The most efficient and low-cost strategy to develop new antifungals involves the redevelopment and utilization of existing clinically approved drugs by clarifying the pharmacological activities, molecular targets, and modes of action [13–15].

Riboflavin (RF), an essential micronutrient for all life forms, is a water-soluble yellow fluorescent substance isolated from milk whey [16]. RF is the core component of the coenzymes flavin adenine dinucleotide and flavin mononucleotide [17]. As a coenzyme of flavin, RF participates in a series of oxidation-reduction reactions and plays key roles in energy production via metabolism of fats, ketone bodies, carbohydrates, and proteins [18,19]. Previous investigations have confirmed the antibacterial [20,21], anti-inflammatory [22], and antioxidant [23] activities of RF. Our group previously reported that endogenous and exogenous RF can effectively prevent *Candida albicans* infection [24,25]. However, it remains unclear whether RF is effect against *C. neoformans*.

Therefore, the aim of the present study was to investigate the potential anti-fungal effect of RF against *C. neoformans* and identify candidate targets to clarify the underlying mechanism.

## Materials and methods

### Strains, chemicals, reagents, and culture conditions

*C*. *neoformans* strain H99 was purchased from the American Type Culture Collection (Manassas, VA, USA), stored in 30% glycerol at −80°C, and cultured overnight in YPD medium (1% yeast extract, 2% peptone, and 2% dextrose) at 37°C and 200 rpm prior to use. Eosin Y triarylmethane dye, 2,3-bis(2-methoxy-4-nitro-5-sulfophenyl)-2 H-tetrazolium-5-carboxanilide (XTT) and menadione were obtained from Shanghai Macklin Biochemical Co., Ltd. (Shanghai, China). Roswell Park Memorial Institute (RPMI) 1640 medium was purchased from HyClone Laboratories, Inc. (Logan UT, USA). RF injection, calcium fluoride white (CFW), sodium dodecyl sulfate (SDS), fluorescein isothiocyanate-labeled wheat germ agglutinin (FITC-WGA), 2,’7’-dichlorodihydrofluorescein diacetate (DCFH-DA), and fluconazole (FCZ) were purchased from Sigma-Aldrich Corporation (St. Louis, MO, USA). FACSAria™ Multi – Analyte Flow Assay Kit was obtained from BioLegend (Beijing) Biotechnology Co., Ltd.

## Antifungal susceptibility assay

In accordance with the Clinical and Laboratory Standards Institute (2017) standard M27-A4, a microdilution broth susceptibility assay was conducted to determine the minimum inhibitory concentration (MIC) of RF against H99 cells. Briefly, overnight cultured H99 cells were resuspended in RPMI-1640 to a final concentration of 2.5 × 10^3^ cells/mL and incubated with RF serially diluted with RPMI-1640 to final concentrations of 0, 0.025, 0.05, 0.1, 0.2, 0.3, 0.4, 0.5, 0.6, 0.7, and 0.8 mg/mL in the wells of 96-well plates along with a yeast-free negative control at 35°C for 72 h. The optical density at 600 nm (OD_600_) was measured using a Varioskan™ LUX multimode microplate reader (Thermo Fisher Scientific, Waltham, MA, USA) to determine the MIC_90_ value (MIC refers to the lowest drug concentration that can inhibit the growth and reproduction of microorganisms; MIC_90_ refers to the MIC required to inhibit 90% of the growth of the tested microorganism).

The medium of four RF treatment wells (RTW) close to the MIC wells in the drug susceptibility test and drug-free positive control wells (PCW) were mixed, diluted with phosphate-buffered saline (PBS), and cultured on YPD solid medium at 37°C for 72 h. Then, the number of viable fungal cells was calculated as colony-forming units (CFUs)/mL. The growth inhibitory factor (IR) was calculated as log(CFUs/mL PCW) − log(CFUs/mL RTW). The minimum fungicidal concentration (MFC) was defined as the lowest concentration of an antifungal resulting in the death of 99.9% of the inoculum (i.e. IR ≥ 3). As described in a previous study [26], an agent with an MFC/MIC ratio ≤4 was considered fungicidal, whereas an agent with an MFC/MIC ratio >4 was considered fungistatic. All assays were performed in triplicate.

## Growth curve and spot dilution assays

As previously reported [27], overnight cultured H99 cells were washed with PBS, resuspended in RPMI-1640 to a final concentration of 10^5^ cells/mL, and incubated with RF at final concentrations of 0, 0.1, 0.2, 0.4, and 0.8 mg/mL in the wells of a 96-well plate, along with cells treated with FCZ (32 μg/mL) as a positive drug control, at 37°C for 72 h. At each time point, the medium in each well was collected and diluted with PBS. Then, the number of CFUs was quantified after incubation at 37°C for 72 h. The above experiments were each performed in triplicate, the assay was performed in triplicate.

As described previously [28], overnight cultured H99 cells were adjusted to 10^2^–10^6^ and cultured with various concentrations of RF on solid medium heated to about 50°C by high-temperature sterilization. After coagulation, 3 μL of the fungal suspension were spotted on YPD plates and cultured at 37°C for 72 h. The plates were photographed using a digital camera (Canon Inc., Tokyo, Japan).

## Melanin and urease production assay

Cryptococcal melanization was assessed using minimal medium (15 mM glucose, 10 mM MgSO_4_, 21.4 mM KH_2_PO_4_, 13 mM glycine, 3 mM thiamine) supplemented with 1 mM L-3,4-dihydroxyphenylalanine (L-DOPA; Sigma-Aldrich Corporation) and 1% agar powder [29]. After heat sterilization, the plates were cooled to 50°C with or without the addition of RF at various concentrations. Overnight cultured H99 cells were adjusted to 1 × 10^8^ cells/mL and serial diluted by 10-fold. Suspended fungal cells (3 μL) were cultured on plates at 37°C for 5 d and photographed.

The ability to secrete urease by fungal cells cultured on minimal medium supplemented with 2% urea and 0.0012% phenol red was assessed [30]. Briefly, overnight cultured H99 cells were resuspended in medium at 2 × 10^6^ cells/mL and further cultured in the wells of 96-well plates with or without various concentrations of RF in urea broth, along with FCZ (32 μg/mL) as a positive control and cell-free medium as a negative control, at 37°C for 48 and 72 h. A microplate reader was used to measure the OD_600_ values and the absorbance of the supernatant (100 μL) at 570 nm.

## Capsule formation and thickness analysis

As described previously [31], capsule formation was induced using medium consisting of 10% sabouraud dextrose broth in 50 mM 3-(N-morpholino) propanesulfonic acid (pH 7.3). Overnight cultured H99 cells (10^6^ cells/mL) were cultured in capsule-inducing medium containing RF (0.1, 0.2, 0.4, and 0.8 mg/mL) at 37°C and 220 rpm for 12 h, along with untreated H99 cells as a control, then centrifuged, washed with PBS, stained with India ink (1:1), and imaged under a bright microscope (model BX63; Olympus Corporation). The capsule size of 50 cells was measured using ImageJ software (https://imagej.net/ij/). Capsule size was defined as the largest distance from the cell wall to the outer edge of the cell.

## Biofilm inhibition and dispersal assay

The biofilm inhibition assay was performed as described previously [32]. Briefly, overnight cultured H99 cells were resuspended in medium at 2 × 10^6^ cells/mL and cultured in the wells of 96-well plates with or without various concentrations of RF in YPD liquid medium, along with cells treated with AMB (0.5 μg/mL) as a positive control, at 37°C for 48 h. The biofilm, which formed on the bottom of the wells, was washed twice with PBS. Then, 200 μL of PBS and 12 μL of XTT-menadione solution (1 mg/mL XTT and 0.4 mM menadione were mixed in a ratio of 5:1) were added to each well and incubation was continued at 37°C for 2 h in the dark. Afterward, the OD_490_ values were measured with a microplate reader. For biofilm dispersion analysis, overnight cultured H99 cells were incubated in the wells of 96-well plates at 37°C for 48 h to induce biofilm formation and rinsed twice with PBS, various concentrations of RF were added and co-cultured for another 48 h. No RF was added to the control wells and blank wells. Detection methods for the metabolic activity of biofilms were the same as described for the biofilm inhibition assay, all assays were performed in triplicate.

## Transmission electron microscopy

Overnight cultured H99 cells were cultured with RF (0.4 and 0.8 mg/mL) or without in YPD liquid medium at 37°C for 6 h, washed with PBS, collected, fixed overnight with 2.5% glutaraldehyde at 4°C, postfixed with 1% osmium tetroxide for 1 h, washed again with PBS, dehydrated with increasing concentrations of ethanol (30%, 50%, 70%, 80%, 90%, and 95%) for 15 min, followed by 100% ethanol for 20 min, embedded in Epon 12 resin (Ted Pella, Inc., Redding, CA, USA), and cut into ultrathin sections (70–90 nm), which were stained with uranyl acetate and lead citrate, and examined with a transmission electron microscope (model 1230; JEOL, Ltd., Tokyo, Japan). The cell wall thickness of at least 20 cells was measured with ImageJ software [29].

## Cell wall assay to confirm contents of chitooligomers, chitin, and chitosan, and response to stress

H99 cells were cultured in YPD liquid medium with RF (0.1, 0.2, 0.4, and 0.8 mg/mL) or without at 37°C and 200 rpm for 6 h, washed with PBS, resuspended to 5 *×* 10^7^ cells/mL, and stained as described previously [33,34]. To detect chitin and chitooligomers, the treated fungal cells were stained with 100 µg/mL FITC-WGA at 37°C for 35 min. Chitin was stained with CFW (5 µg/mL) at 37°C for 10 min, chitosan was stained with Eosin Y at 37°C for 10 min, and β-1,3-glucan was stained with 0.1% aniline blue (Wako Pure Chemical Industries, Ltd., Osaka, Japan) at 80°C for 15 min in the dark. The stained cells were washed, resuspended in PBS, and observed at 40 × under a fluorescence microscope (model BX63; Olympus Corporation). Fluorescence was measured at excitation and emission wavelengths of 400 and 460 nm, respectively, to determine the total glucan content and 365 and 435 nm to determine the total chitin content.

For analysis of cell stress, the RF-treated cells were resuspended in PBS at 1 × 10^7^ cells/mL, serially diluted, and cultured on YPD agar containing CFW, SDS, or hydrogen peroxide at 37°C for 72 h.

## Propidium iodide (PI) staining and cell membrane permeability assay

Overnight cultured H99 cells were cultured with RF (0.4 and 0.8 mg/mL) or without for 6 h, then resuspended in PBS at 10^7^ cells/mL. Cell membrane permeability was assessed by staining with 10 mg/mL of PI (Solarbio Science and Technology Co., Ltd., Beijing, China) at 37°C for 30 min in the dark as described previously [25,35]. After washing and resuspension in PBS, membrane permeability was assessed using a flow cytometer (BD FACSAria™ III Cell Sorter; BD Biosciences, Franklin Lakes, NJ, USA) and an inverted fluorescence microscope. An untreated group was included as a blank control.

## Production of reactive oxygen species (ROS)

Overnight cultured H99 cells were cultured with RF (0.4 and 0.8 mg/mL) or without for 6 h and then resuspended in PBS to 10^7^ cells/mL. The intracellular ROS content was determined by staining with 10 μM DCFH-DA at 37°C for 30 min in the dark [36]. After washing and resuspension in PBS, the intracellular ROS content was measured using a cytometer, a multifunctional microplate reader (excitation and emission wavelengths of 485 and 530 nm, respectively), and an inverted fluorescence microscope. An untreated group was included as a blank control.

## RNA isolation and reverse transcription quantitative polymerase reaction (RT-qPCR)

H99 cells were cultured in YPD liquid medium with 0.4 mg/mL of RF or without at 37°C for 6 h with shaking (200 rpm) and collected by centrifugation. Then, total RNA was isolated using yeast processing reagent (Takara Biotechnology Dalian Co., Ltd., Dalian, China) and reverse transcribed with a PrimeScript™ RT reagent Kit with gDNA Eraser (Takara Biotechnology Dalian Co., Ltd.) into complementary DNA, which was amplified by RT-qPCR with TB Green® Premix Ex Taq™ II master mix (Takara Biotechnology Dalian Co., Ltd.) and the primers listed in Table S1. Relative changes to gene expression levels were determined using the 2^−ΔΔCt^ method and normalized to expression of glycerol-3-phosphate dehydrogenase 1 [37], the assay was performed in triplicate.

## Evaluation of antifungal activity in vivo

The protocol of the animal study was approved by the Institutional Animal Care and Use Committee of Southwest Medical University (approval 20,210,927–021) and conducted in accordance with the guidelines of the Guide for the Care and Use of Laboratory Animals. Before the experiment, H99 cell growth in YPD medium at 37°C and 200 rpm for 16 h, washed, and resuspended in normal saline. Female C57BL/6J mice (age, 6–8 weeks) were obtained from Sipeifu (Beijing) Biotechnology Co., Ltd. (Beijing, China). All the experimental animals were housed in an animal care facility with libitum access to food and water. A mouse model of pulmonary cryptococcosis was generated as previously described [38]. The mice were randomly assigned to each control group and treatment group using an Excel table and cage positions are randomly arranged. The operation and sampling sequences are disrupted, and the control variables are controlled. During the experiment, animals exhibited a body weight loss exceeding 20%, severe motor impairment, or persistent refusal to eat, humane euthanasia was performed (cervical dislocation after anesthesia with pentobarbital 50 mg/kg). The mice were anesthetized with 5% isoflurane until the heart beat slowed to about 1 beat per second. Once anesthetized, 50 μL of 10^6^ cells/mL were dripped onto the nares of mice (5 mice per group), so that each mouse inhaled the full inoculum. At 2 h post-infection, mice in the treatment group were intraperitoneally injected with RF (1 or 2 mg/kg) or FCZ (12 mg/kg), while mice in the control (non-infection) and Cn (infection) groups were intraperitoneally injected with normal saline. The mice were injected once every 24 h for 7 consecutive days. Blood samples were collected for cytokine analysis, and partial lung tissues were collected for pathological analysis. The remaining lung tissues and brain tissues were homogenized, serially diluted with PBS, and 100 μL aliquots were inoculated on YPD agar containing 100 μg/mL of ampicillin and streptomycin at 37°C for 72 h. Resulting yeast colonies were counted.

Female Institute of Cancer Research mice (age, 3–4 weeks) were obtained from Sipeifu (Beijing) Biotechnology Co., Ltd. To generate a mouse model of disseminated cryptococcosis, the mice were tail vein injection injected with 100 μL of suspended fungal cells (10^5^ cells/mL). The experimental group, dosage, and mode of administration were the same as in the previous pulmonary infection model; however, the mice were injected once every 24 h for 3 or 7 consecutive days. Afterward, the mice were humanely euthanized and blood samples were collected for cytokine analysis, while partial brain, liver, and kidney tissues were collected for pathological analysis. The remaining brain, lung, liver, and kidney tissues were homogenized, serially diluted with PBS, and 100 μL aliquots were inoculated on YPD agar and incubated at 37°C for 72 h. Resulting yeast colonies were counted.

The Cytometric Bead Array (CBA) method was used to detect the levels of relevant cytokines (including IFN-γ, TNF-α, IL-4, IL-10, and IL-6) in mouse plasma. The analysis was carried out through multiple steps, including kit setup, incubation with plasma samples, washing and detection antibody addition, and final washing and data acquisition. The data were then analyzed using a flow cytometer (BD FACSAria™ III Cell Sorter; BD Biosciences, Franklin Lakes, NJ, USA).

## Effects of RF on proliferation of MH-S macrophages and intracellular ROS levels

100 uL suspended MH-S cells (1 × 10^5^ cells/mL), a murine alveolar macrophage cell line, were added to the wells of a 96-well plate and cultured in DMEM- (Dulbecco’s Modified Eagle Medium-) high glucose supplemented with 10% fetal bovine serum, 100 U/mL penicillin and 100 μg/mL streptomycin, incubated overnight at 37°C under an atmosphere of 5% CO_2_/95% air. Various concentrations of RF (5–160 μg/mL) were added to the appropriate wells. The control group and cell-free blank group were set in the 96-well plate and incubated for 2 h and 4 h. After removal of the upper layer of the medium, the cells were washed twice with 1 × PBS. Then, 100 μL of 10% cell counting kit-8 reagent (APExBIO Technology, Houston, TX, USA) was added to each well and incubation was continued for 2 h [15]. The OD was measured at 450 nm using a microplate reader. The cell survival rate (%) was calculated as (OD administration − OD blank)/(OD control − OD blank) × 100%.

After the addition of various concentrations of RF (0–40 μg/mL), 100 uL MH-S cells (2 × 10^6^ cells/mL) were incubated for 2 h. Afterward, the upper medium was removed, and the cells were washed twice with PBS. DCFH-DA was then added to the culture medium at 10 μmol/L. A group without DCFH-DA staining solution was included to account for background fluorescence. These cells were incubated for 20–30 min. After incubation, the cells were washed three times with Dulbecco’s modified Eagle’s medium (DMEM; Shanghai Yuanpei Biotechnology Co., Ltd., Shanghai, China). The fluorescence intensity was measured with a microplate reader at excitation and emission wavelengths of 488 and 525 nm, respectively [39].

## Effects of RF on phagocytosis and killing of C. neoformans by MH-S macrophages

100 μL MH-S macrophages (2 × 10^6^ cells/mL) were cultured overnight in the wells of 96-well plates. After removal of the upper medium, RF at 0, 10, 20, and 40 μg/mL was added to the wells and incubation was continued for 2 h. H99 cells were washed and resuspended in DMEM medium to stimulate MH-S cells for 2 h (phagocytosis ratio, 1:5) [29]. Phagocytosis was terminated by the addition of ice-cold PBS, and the unphagocytosed cryptococcal cells were removed by washing. Then, 200 μL of ultra-pure water were added to each well to lyse the cells. Subsequently, 100 μL aliquots of the cells were evenly coated on YPD ager and incubated at 37°C for 72 h. Afterward, the number of CFUs was counted.

To investigate the killing effect of macrophages pretreated with RF on fungi, after pretreatment with RF and phagocytosis of fungi as described above, the cells were washed with PBS to terminate phagocytosis and remove extracellular *Cryptococcus neoformans* that had not been phagocytosed. Subsequently, fresh DMEM medium was added, and the cells were cultured for 24 h. Finally, the cells were lysed, and the content was spread on YPD plates and incubated at 37°C for 72 h. Then, the number of CFUs was counted [40], all assays were performed in triplicate.

The above experimental methods followed the ARRIVE guidelines. https://arriveguidelines.org/sites/arrive/files/documents/Author%20Checklist%20-%20Full.pdf.

## Statistical analysis

All experiments were repeated three times. All statistical analyses were performed using Prism 9.0 software (GraphPad Software, LLC, San Diego, CA, USA). As the data were normally distributed, differences between the groups were compared with the *t*-test, log-rank test, or one-way analysis of variance (ANOVA). A probability (*p*) value <0.05 was considered statistically significant.

## Results

### Antifungal activity of RF against C. neoformans in vitro

**Figure 1.**
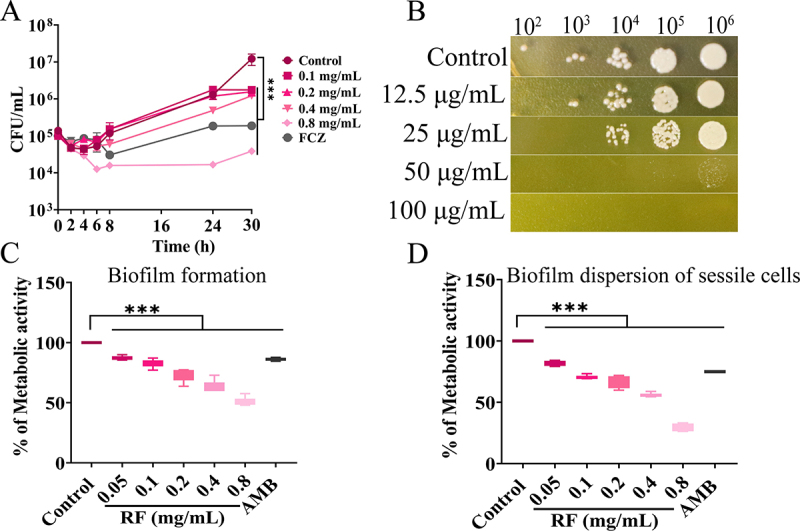
Antifungal activity of RF against *C. neoformans in vitro*. (A) growth curve of *C. neoformans* (initial inoculum of 2 × 10^5^ CFU/mL) treated with RF. FCZ, fluconazole (32 μg/mL). (B) spot dilution assay on solid medium containing various concentration of RF. Metabolic activity of *C. neoformans* biofilms was determined with the 2,3-bis-(2-methoxy-4-nitro-5-sulfophenyl)-2 H-tetrazolium-5-carboxanilide assay. (C) effect of RF on biofilm formation (48 h). (D) effect of RF on the viability of sessile cells from mature biofilms (preformed 48 h), AMB, amphotericin B (0.5 μg/mL). Results represent average % metabolic activity ± SD. Data were analyzed by one-way ANOVA (***p < 0.001).

The MIC of RF against *C*. *neoformans* was 0.4 mg/mL, while the MFC was 0.8 mg/mL. In addition, the growth curve assay (Figure 1A) showed that RF significantly inhibited the growth of *C. neoformans* at different time points, and treatment with RF at 0.4 mg/mL for 24 h achieved 90% fungal inhibition efficiency. Furthermore, *C. neoformans* exhibited severe growth defects on solid media supplemented with 50 µg/mL RF (Figure 1B), specifically, at an identical fungal concentration, a remarkable reduction in the number of fungal cells was observed, accompanied by a sparse distribution pattern. Additionally, the size of individual fungal cells was notably diminished. *C. neoformans* biofilm is a complex community structure that can form on polystyrene plates and medical devices, which can result in chronic infection [41]. Here, RF (0.05–0.8 mg/mL) significantly inhibited biofilm formation (Figure 1C and S1-A) and effectively reduced the viability of sessile cells from mature biofilms (Figure 1D and S1-B).

## RF caused structural damage to yeast cells and reduced virulence

**Figure 2.**
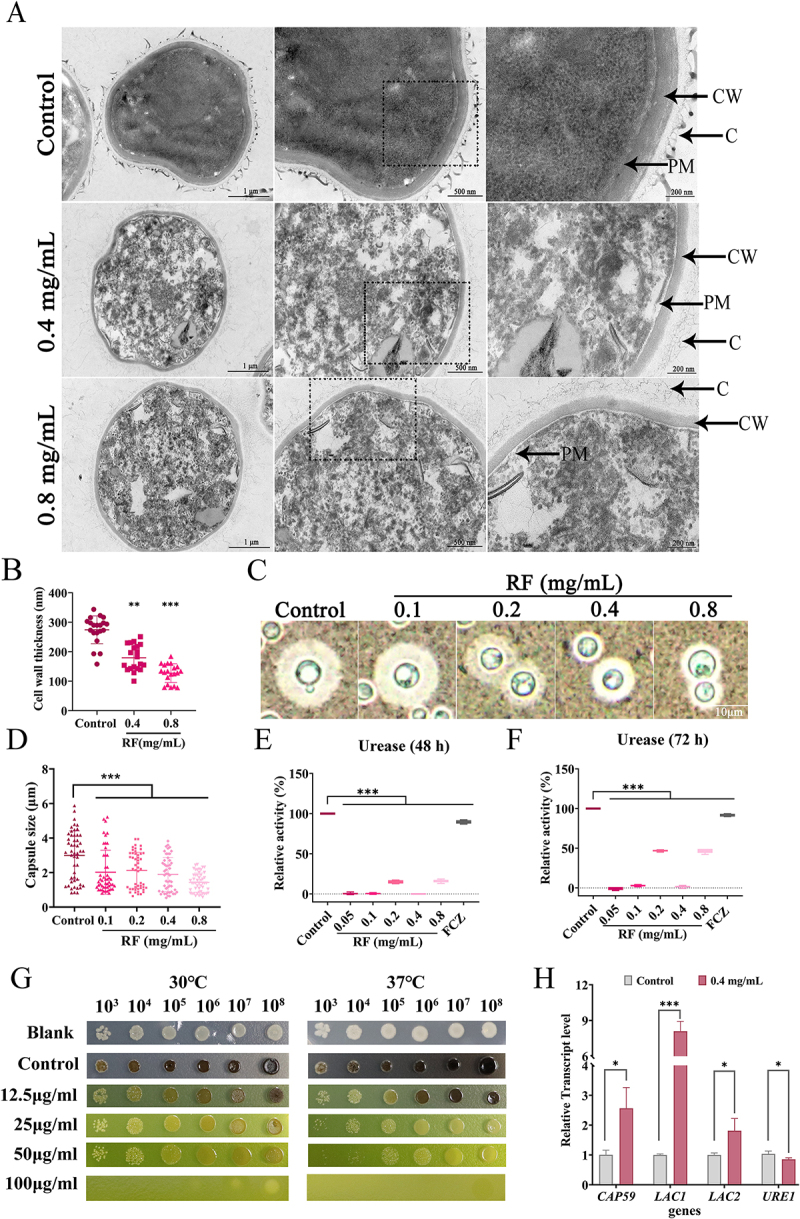
Effect of RF on the cellular structure of *C. neoformans*. (A) transmission electron microscopy of *C. neoformans* structures after RF treatment. Bar: 1 μm, 500 nm, or 200 nm. PM, plasma membrane; CW, cell wall; C, capsular material (B) determination of cell wall thickness (20 cells per group). (C) India ink staining of the capsule of *C. neoformans* at 12 h of RF treatment. (D) capsule size measurements of the control and RF-treated *C. neoformans*. The capsule size of 50 cells was measured in each group. (E, F) urease activity assay of *C. neoformans* was performed using urea broth medium supplemented with phenol red at 48 and 72 h, 32 μg/mL fluconazole was used in this assay. (G) melanin production of *C. neoformans* was assessed at 30°C and 37°C in micromedia supplemented with L-DOPA for melanin induction and RF. (H) transcript levels of genes related to virulence factors quantified by RT-qPCR and normalized to glycerol-3-phosphate dehydrogenase 1. Data were analyzed by one-way ANOVA and the *t*-test (*p < 0.05; **p < 0.01; ***p < 0.001).

Transmission electron microscopy showed that RF-treated *C. neoformans* cells exhibited uneven cytoplasm, sparse capsules, and discontinuous plasma membranes (Figure 2A). In addition, the cell membrane was separated from the cell wall and the width of the fungal cell wall layer was shortened (Figure 2B). Subsequently, the effect of RF on virulence factors were explored. As shown in Figures 2C and 2D, RF significantly reduced the capsule size of *C. neoformans* cultured in capsule-induction medium. In urea medium supplemented with phenol red, RF significantly inhibited urease activity of *C. neoformans* at 48 h and 72 h (Figure 2E and 2F). Besides, melanin production of *C. neoformans* was decreased in L-DOPA melanin-induction medium containing RF (Figure 2G). Meanwhile, the RT-qPCR results showed that RF significantly reduced expression of the urease synthesis gene (*URE1*) and increased expression of genes related to capsule formation (*CAP59*) and melanin synthesis (*LAC1* and *LAC2*) (Figure 2H).

## RF induced damage to the cell wall and activated the cell wall damage repair pathway

**Figure 3.**
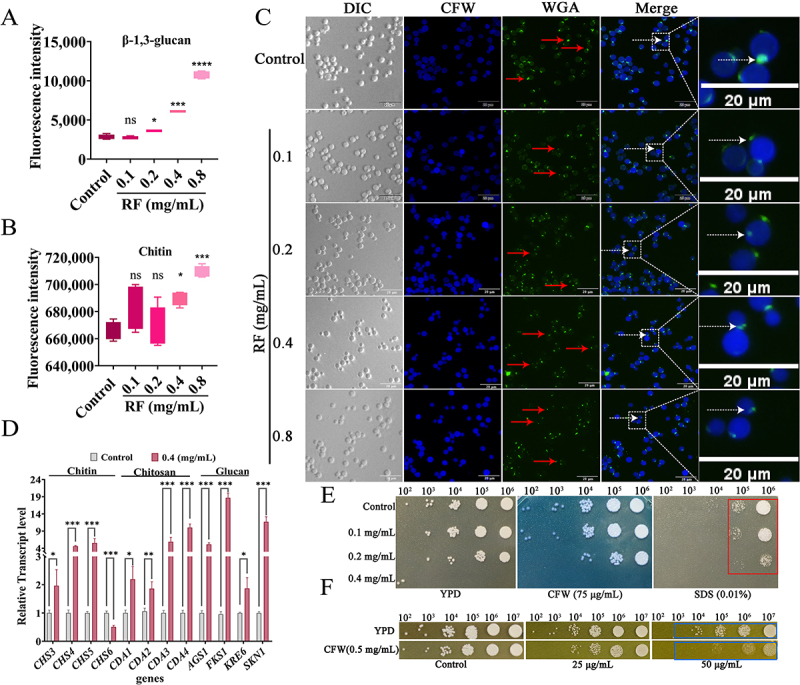
RF induced damage to the cell wall of *C. neoformans*. Determination of (A) total glucan and (B) chitin in fungal cell walls after RF treatment. (C) analysis of chitin (stained with CFW) and chitooligomers (stained with FITC-WGA) in fungal cell walls after RF treatment, and photographed under an inverted fluorescence microscope. The red and white arrows represent a decrease in the exposure of chitosan oligomers. Bar: 20 μm. (D) expression of genes involved in cell wall biosynthesis as determined by RT-qPCR analysis. *C. neoformans* treated with RF and diluted continuously in plates with or without (E) 75 μg/mL of CFW, 0.01% SDS, and (F) 0.5 mg/mL of CFW. Red and blue boxes indicate growth defects of RF-treated *C. cryptococcus* on plates containing cell wall/surface stressors. Data were analyzed by one-way ANOVA and the *t*-test (ns, *p* > 0.05; *p < 0.05; **p < 0.01; ***p < 0.001; ****p < 0.0001).

RF increased the *C*. *neoformans* cell wall contents of β-1, 3-glucan (Figure 3A), chitin (Figure 3B), and chitosan (Fig. S2-A). Furthermore, RF reduced formation of chitooligomers (red and white arrows) (Figure 3C). The results of RT-qPCR analysis showed that RF significantly up-regulated expression of genes encoding chitin synthase (*CHS3*, *CHS4*, and *CHS5*), chitin deacetylase (*CDA1*, *CDA2*, *CDA3*, and *CDA4*), and those associated with cell wall biosynthesis (*FKS1*, *AGS1*, and *SKN1*) and glucan synthesis (*KRE6*), while *CHS6* was significantly down-regulated (Figure 3D). In addition, RF significantly up-regulated expression of three genes related to cell wall integrity (*Rho1*, *Pkc1*, and *Mpk1*) (Fig. S2-B). Moreover, the results of the stress assays further verified that RF impaired cell wall integrity (Figure 3E and 3F) (red and blue boxes). These results suggest that RF caused damage to the cell integrity of *C. neoformans* and induced cell wall remodeling via activation of the cell wall integrity pathway.

## RF altered the cell membrane integrity of C. neoformans

**Figure 4.**
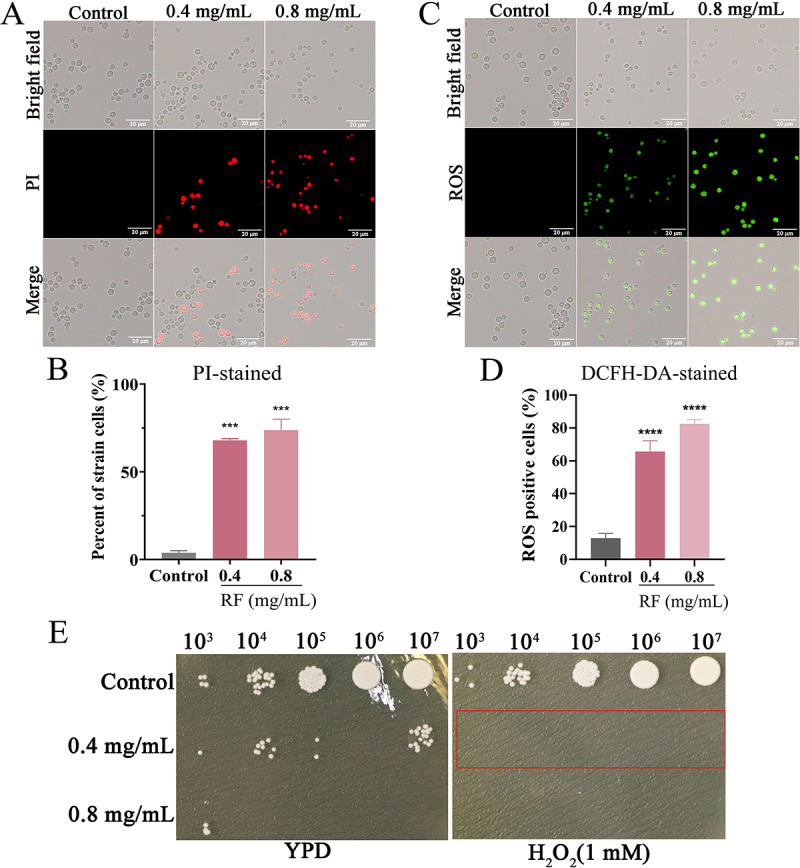
Effect of RF on cell membrane integrity and intracellular accumulation of ROS. Cells were photographed under a fluorescence microscope (A) and analyzed by flow cytometry (B). PI staining of the damaged cell membrane (red fluorescence). Bar: 20 μm. Intracellular ROS levels were assessed by fluorescence microscopy (C) and flow cytometry (D). Intracellular ROS were labeled with DCFH-DA (green fluorescence). Bar: 20 μm. (E) hydrogen peroxide was added to YPD plates to evaluate the sensitivity of *C. neoformans* to oxidative stress. Red box, growth defects of *C. cryptococcus* after addition of RF. Data were analyzed by one-way ANOVA (***p < 0.001; ****p < 0.0001).

As shown in Figure 4A, RF disrupted the cell membrane of *C*. *neoformans*, as a potential mechanism of fungal inhibition. Staining with PI was performed to explore the effects of RF on the membrane of *C. neoformans* cells, red fluorescence was significantly increased after RF treatment. Similarly, the flow cytometry results further confirmed that RF at 0.4 and 0.8 mg/mL increased the percentage of PI-stained cells to 74.62% and 89.51%, respectively (Figure 4B and S3-A). These results demonstrated that RF effectively killed *C*. *neoformans* cells via damage to the cell membrane and enhanced cell permeability.

## RF induced intracellular accumulation of ROS

ROS are involved in various metabolism-related intracellular activities, although excessive production of intracellular ROS causes metabolic damage [42]. Thus, ROS production was measured. As shown in Figure 4C, as compared to the control group, green fluorescence intensity was significantly increased after RF treatment. The flow cytometry results showed that RF at 0.4 and 0.8 mg/mL increased production of ROS by 72.30% and 84.06%, respectively (Figure 4D and S3-B), indicating that RF caused intracellular accumulation of ROS. Furthermore, the growth of RF-treated cells was restricted on plates supplemented with 1 mM H_2_O_2_ (Figure 4E) (Red box). Hence, RF induced accumulation of intracellular ROS and increased sensitivity to oxidative stress.

## RF reduced the fungal burden and inflammation in mouse models of lung infection and CM

**Figure 5.**
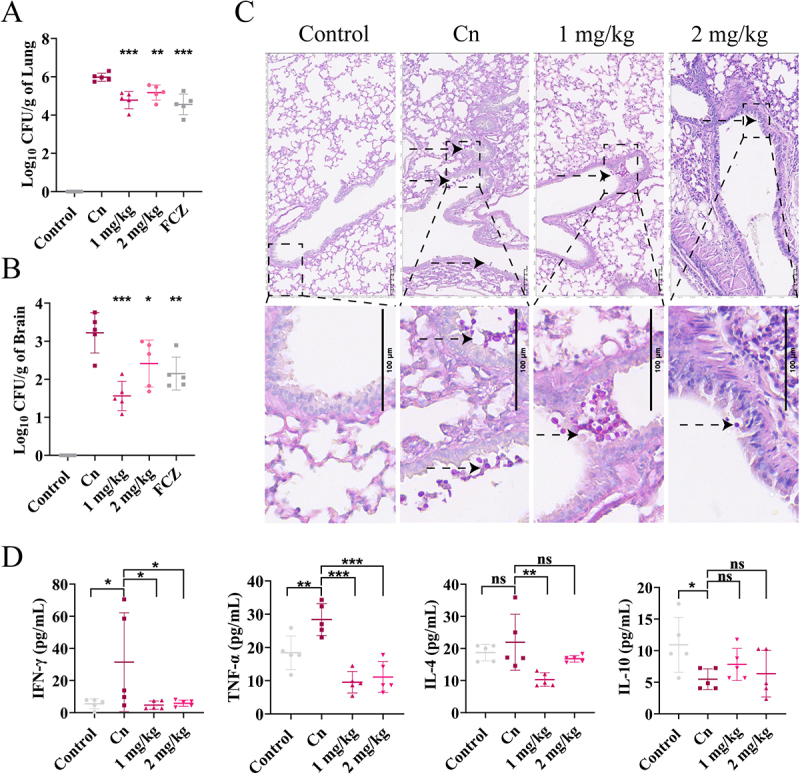
Efficacy of RF against intranasal infection *in vivo*. Determination of fungal load in lung (A) and brain (B) tissues of mice 7 d after infection. (C) pathological analysis of lung tissue (stained with PAS). Black arrows, *C. neoformans*. Bar: 100 μm. (D) plasma cytokine levels were assessed by flow cytometry. Data were analyzed by one-way ANOVA (ns, *p* > 0.05; *p < 0.05; **p < 0.01; ***p < 0.001).

An intranasal model of cryptococcal infection was used to mimic the natural course of infection, subsequent pulmonary infection, and dissemination to other organs, especially the brain. In a mouse model of intranasal infection (Fig. S4), as compared to the Cn group, the fungal burden in the lungs and brain was significantly reduced in the RF group (Figure 5A and 5B). To assess the curative efficacy of RF *in vivo*, histopathological changes of the lungs were evaluated by periodic acid-Schiff (PAS) staining. RF inhibited fungal proliferation near the main bronchi (black arrow) (Figure 5C). Meanwhile, plasma cytokine analysis showed that as compared to the Cn group, the levels of the proinflammatory cytokines IFN-γ and TNF-α were significantly reduced, along with expression of the anti-inflammatory cytokine IL-4, after treatment with RF at 1 or 2 mg/kg, while there was no change to IL-10 levels (Figure 5D).

**Figure 6.**
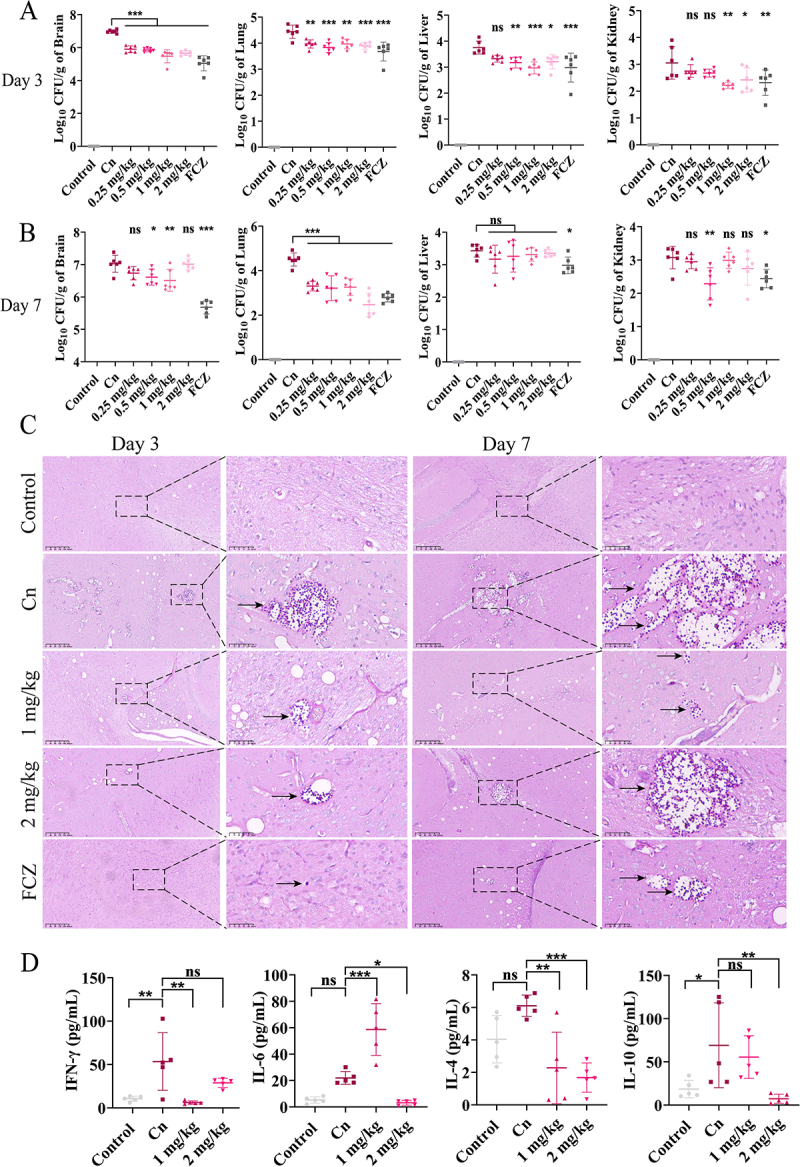
Efficacy of RF against intravenous infection *in vivo*. Determination of fungal load in brain, lung, liver, and kidney tissues of mice after 3 (A) and 7 (B) d. (C) pathological analysis of brain tissue (stained with PAS). *C. neoformans*. Black boxes, site of lesion. Bar: 200 μm or 50 μm. (D) flow cytometry of plasma cytokine levels of mice at 3 d after infection. Data were analyzed by one-way ANOVA (ns, *p* > 0.05; *p < 0.05; **p < 0.01; ***p < 0.001).

Subsequently, the efficacy of RF against intravenous infection was further evaluated (Fig. S5). As compared to the Cn group, RF at 1 or 2 mg/kg for 72 h significantly reduced the fungal burden in the brain, lungs, liver, and kidneys (Figure 6A). Importantly, RF at 0.5 mg/kg after 7 d still significantly reduced infection of the brain, lungs, and kidneys of mice, although there was no significant difference in the liver (Figure 6B). Staining with PAS and hematoxylin-eosin (H&E) showed that RF decreased the fungal burden (purple particles, black arrows) and the severity of infective lesions (black boxes) in brain tissue (Figure 6C and S6), and significantly reduced the fungal load in the kidneys (purple particles, black arrows) (Fig. S7), but not the liver. Moreover, RF attenuated inflammatory cell infiltration in the blood vessels of the liver tissue (black arrows) (Fig. S8). In addition, as compared to the Cn group, RF at 1 and 2 mg/kg significant decreased plasma levels of the cytokines IL-4 and IL-6. In addition, expression of the cytokine IFN-γ was significantly decreased by RF at 1 mg/kg RF, and plasma expression of IL-10 was significantly increased by RF at 2 mg/kg (Figure 6D).

## Interaction among RF, MH-S alveolar macrophages, and C. neoformans

**Figure 7.**
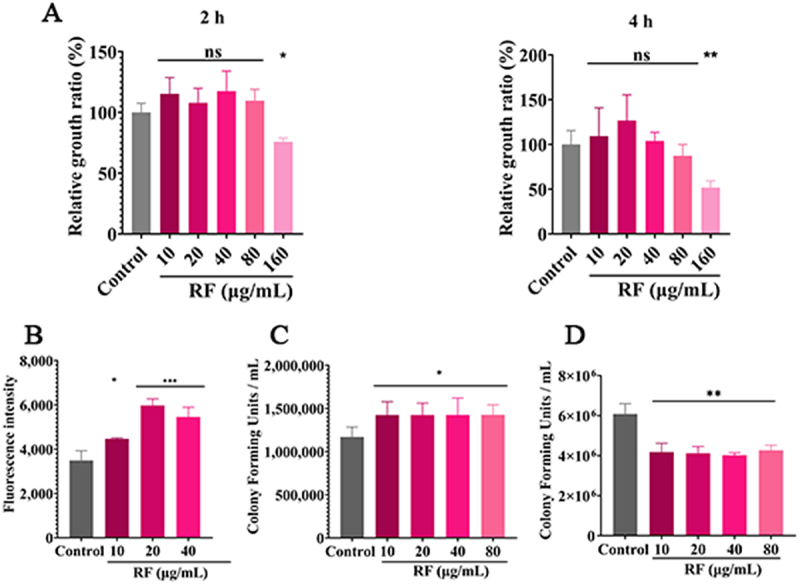
RF promoted phagocytosis and killing of *C. neoformans* by macrophages. (A) the proliferation activity of MH-S macrophages was determined with the cell counting kit-8 assay at 2 and 4 h. (B) ROS levels in MH-S cells were evaluated with a microplate reader. The effect of RF on the phagocytosis (C) and killing (D) abilities of MH-S cells (phagocytosis ratio, 1:5) was investigated. Data were analyzed by one-way ANOVA (ns, *p* > 0.05; *p < 0.05; **p < 0.01; ***p < 0.001).

The results of the proliferation activity assay showed that RF at 80 μg/mL was not significantly toxic to MH-S cells at 2 and 4 h (Figure 7A). Moreover, there was no significant effect on cell morphology by microscopic observation (Fig. S9-A). In addition, RF treatment significantly increased intracellular ROS levels in MH-S cells (Figure 7B). Meanwhile, RF treatment significantly promoted phagocytosis and killing of *C*. *neoformans* by MH-S cells (Figure 7C and 7D). Collectively, these findings revealed that RF promoted the phagocytic and killing effects of MH-S cells.

## Discussion

**Figure 8.**
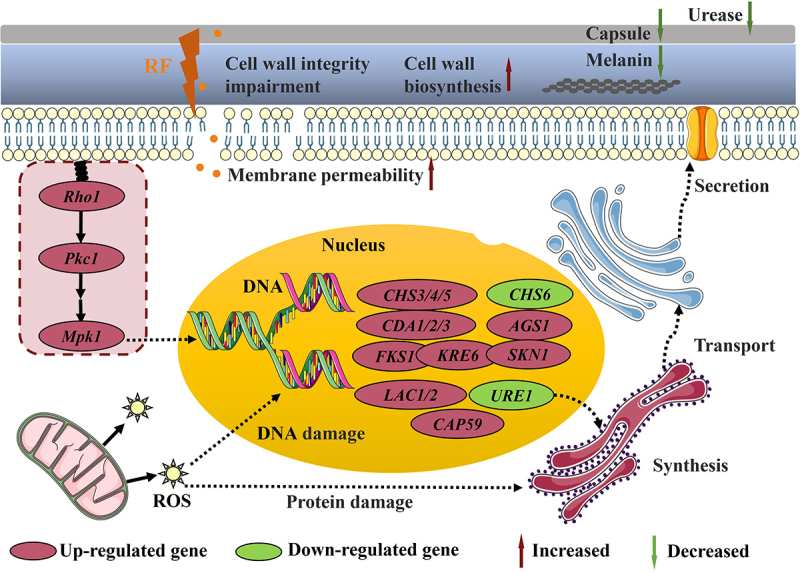
The main antifungal effects of RF and adaptive regulatory effects of *C. neoformans*. RF damaged the cell membrane, inhibited melanin synthesis and urease secretion, and decreased the capsule size. The accumulation of ROS induced metabolic damage and further caused fungal death. Meanwhile, RF compromised cell wall integrity, triggered the cell wall damage and repair signaling pathway, up-regulated expression of genes related to the Mpk1 pathway, and regulated the expression of genes associated with cell wall biosynthesis (*CHS3*, *CDA1*, and *FKS1*) and virulence (*CAP59*, *Lac1*, *Lac2*, and *Ure1*). Fungal adaptation modulated the *C. neoformans* cell wall structure. Red ovals, up-regulation; green ovals, down-regulation.

Aspiration of dry yeast or basidiomycetes from contaminated soil or guano is considered the main route of *C. neoformans* infection in humans [43]. *C*. *neoformans* mainly causes deep fungal infections of the lung and brain, which can be life-threatening in severe cases. At present, highly drug-resistant strains of *C*. *neoformans* pose serious risks to human health [10,44]. Current treatment strategies for *C*. *neoformans* infection mainly include combination therapy, new use of old drugs, targeted virulence reduction, regulation of host immunity (vaccine development), and inhibition of the stress response [45]. The development of antifungal drugs has continued to rapidly progress. However, many new antifungal agents are still in the clinical trial stage (e.g. APX001 and tamoxifen) or basic research stage (e.g. benzothioureas and clofazimine). Drug repurposing is the exploration of new uses of existing approved drugs, repositioning of pharmacological effects and molecular targets [14,26], and improving drug potency through selective optimization. For example, vitamin D_3_ is widely used to regulate calcium and phosphate metabolism to promote bone health. A previous study by our group confirmed the significant anti-cryptococcal effects of vitamin D_3_ [27]. RF is an essential vitamin that is synthesized by the skin or supplemented. Recent investigations have shown that adequate dietary and supplementary RF can protect against sepsis, ischemia, and various cancers [23]. In addition, RF is effective for the treatment of migraine and cataracts [46]. Moreover, as shown in Figure 8, the antifungal mechanism of RF primarily involves targeting virulence, disrupting cell membrane integrity, and ROS accumulation, thereby offering additional insights into the therapeutic effects of RF.

Biofilm enables yeast to proliferate in host cells, while the phenotypic and functional characteristics protect the fungus from immune attack [47]. Biofilm produced by *C. neoformans* is resistant to antimicrobial agents and host defenses [48]. Although common antifungal agents are effective against a wide spectrum of yeast, most are not very effective against chronic infections caused by *C. neoformans* biofilm, resulting in increased severe toxicity and even mortality [48,49]. In this study, RF exhibited significant fungicide activities against *C. neoformans* (planktonic cells, biofilm formation, and preformed biofilm) (Figure 1), demonstrating the efficacy of RF as a fungicidal agent.

Antitoxic agents reportedly reduce evolutionary pressure underlying drug resistance [50]. The development of anti-toxic agents targeting virulence is an effective strategy for treatment of fungal infections. The *Cryptococcus* polysaccharide capsule modulates host immune responses, enhances pathogenicity, and confers protection against oxidative stress [51]. The relatively small capsule of *C. neoformans* reduces resistance to the external environment and is easily recognized and eliminated by the host immune response *in vivo*. Besides, as an important virulence factor of *C. neoformans*, melanin is formed by the substrates of diphenolic compounds, such as L-DOPA [52]. Reduced synthesis of melanin may lead to decreased resistance to various stress factors, including free radicals and heat [53]. In addition, melanin synthesis is dependent on the enzyme bisphenol oxidase, which is encoded by *LAC1* and *LAC2* [54]. Expression of genes related to capsule and melanin biosynthesis was significantly up-regulated (Figure 2H), which appears to be a transcriptional compensation and adaptation mechanism in fungal cells after drug treatment. RF does not directly inhibit capsule and melanin biosynthesis, but directly damages the capsule structure and inhibits binding of laccase to its substrate and deposition into the cell wall. Meanwhile, urease and melanin together regulate the virulence of *C. neoformans* [8]. Urease, a secretase encoded by *C. neoformans URE1*, is also an important virulence component associated with blood–brain barrier penetration. Decreased urease secretion in *C. neoformans* also affects melanin production by regulating environmental pH [55]. Therefore, RF also has broad research potential as an antitoxic agent.

Currently available anti-*C. neoformans* drugs mainly focus on destroying the cell membrane and miscoding of RNA leading to inhibition of DNA synthesis [56]. Drugs targeting the cell wall of *cryptococci* for clinical use are scarce or almost non-existent [57]. RF rapidly up-regulated transcription of genes related to synthesis of cell wall components (Figure 3D and S2-B), suggesting that RF may act directly on the cell wall structure and integrity, followed by triggering pathways that participate in cell wall repair. This further causes cell wall remodeling, as increased exposure to total chitin in the cell wall allows *C. neoformans* to bind more CFW, which manifests *in vitro* as growth restriction. Moreover, exposure to β-1, 3-glucan reduces thickness of the cell wall layer, thereby enabling *C. neoformans* to be recognized and cleared more quickly by the host immune system *in vivo* [58]. Meanwhile, RF disrupted the structural integrity of the cell membrane phospholipid bilayer, resulting in significant cell membrane breakage and destruction (Figure 2A). However, it remains unclear whether RF influences cell membrane biosynthesis. Furthermore, excessive accumulation of ROS can disrupt lipid metabolism, energy metabolism, and protein transport [59]. However, the accumulation of ROS also renders cells more sensitive to various stressors. Structural damage mainly caused by cell membrane destruction and ROS accumulation further accelerate the damage and death of *C. neoformans*. Moreover, the combination of RF and fluconazole exhibited additive or uncorrelated effects (data not shown), possibly because RF and fluconazole target the same or similar molecules.

In the early stage of intranasal infection, RF restricted replication and survival of *C. neoformans* cells in the lung bronchi. RF treatment reduced fungal titers in lung tissue by almost 1 log unit and in brain tissue by almost 2 log units (Figure 5A and 5b). This suggests that RF plays an important role in inhibiting the transmission of *C. neoformans* from the lung to the brain. Moreover, as protective effects, RF targets virulence factors that induce damage to the host cell in the disease process [53]. Therefore, RF greatly mitigates infection by reducing virulence. In addition, RF in the blood can penetrate the blood–brain barrier and directly inhibit fungal replication in brain tissue (Figure 5C). However, further experiments are needed to verify and evaluate the ability of RF to penetrate the blood–brain barrier *in vivo*. Furthermore, in the CM model, RF reduced the fungal load of the lung tissue by 0.5 log units on day 3 and by 1 log unit on day 7 (Figure 6A and 6B), but had no significant effect in other tissues. Therefore, RF may be more conducive to long-term intervention and treatment of pulmonary infection. In both models, the Cn group had significantly higher levels of proinflammatory cytokines (IFN-γ, TNF-α, and IL-6), which are established markers of acute inflammation and induce protective immune responses in mice [60]. Interestingly, the fungal load was reduced along with the host immune response, as demonstrated by lower plasma levels of inflammatory cytokines in the RF treatment group (Figure 5D and 6D). RF exhibits anti-inflammatory activities [61] by effectively reducing release of pro-inflammatory cytokines, thereby alleviating the inflammatory response of tissues and inhibiting migration of fungi across the blood–brain barrier [62]. Moreover, RF reduced levels of anti-inflammatory cytokines and promoted clearance of fungi via regulation of the host immune responses. Alveolar macrophages are the first line of defense against *C. neoformans* [63]. In this study, ROS levels in macrophages were increased after RF treatment (Figure 7B). Continuous oxidative bursts within the phagosome can effectively kill microorganisms [64]. In addition, reduced melanin production inhibits the ability of *C. neoformans* to defend against oxidative damage. Moreover, decreased urease secretion caused by RF partially reduced interference of phagosome acidification and further promoted fungal clearance by macrophages [65]. Further antifungal effects of RF at 20 μg/mL include promotion of pinocytosis activity of MH-S cells (Fig. S9). Collectively, these results show that RF can modulate host immune responses and exert significant antifungal effects [24].

Regarding safety, RF at 1–2 mg/kg caused no significant pathological damage to the liver or kidneys (Fig. S10). In addition, the results of a previous study by our group found that RF at 600 μg/mL was not overly toxic to HepG2 cells within 48 h (data not shown). Lack of RF caused perioral and ocular inflammation, resulting in biological oxidation and metabolic disorders. The dose of RF used in the *in vivo* study was based on clinical dosages for oral, subcutaneous, and intramuscular administration for humans. Meanwhile, RF is absorbed in the small intestine and excreted from the kidneys. Due to limited absorption in the small intestine, a moderate amount of RF is not toxic. Furthermore, RF at 80 μg/mL did not inhibit proliferation or toxic injury of MH-S alveolar macrophages (Figure 7A). These results confirm that RF is safe and effective *in vivo*.

## Conclusion

As an effective antifungal agent against *C. neoformans* infection both *in vitro* and *in vivo*, RF regulates inflammation by reducing secretion of cytokines. The antifungal mechanism of RF is related to membrane disruption, compromised cell wall integrity, and ROS accumulation. Further investigations are needed to explore the antifungal effects of RF *in vivo* as a potential clinical agent against pathogenic fungi.

## Supplementary Material

FigS9.tif

FigS7.tif

FigS2.tif

Supplementary_Material (1).docx

FigS3.tif

FigS4.tif

FigS8.tif

Fig S1.tif

FigS6.tif

Fig S10.jpg

FigS5.tif

Table S1.xlsx

## Data Availability

The data that support the findings of this study are openly available in figshare (https://figshare.com) at https://doi.org/10.6084/m9.figshare.27486108, reference number 27,486,108.

## Notes

No potential conflict of interest was reported by the authors.

## References

[1] Kronstad JW, Attarian R, Cadieux B, et al. Expanding fungal pathogenesis: cryptococcus breaks out of the opportun istic box. Nat Rev Microbiol. 2011;9(3):193–18. doi: 10.1038/nrmicro2522 21326274 PMC4698337

[2] Alanio A, Vernel-Pauillac F, Sturny-Leclère A, et al. *Cryptococcus neoformans* host adaptation: toward biological evidence of dormancy. MBio. 2015;6(2):ee02580–14. doi: 10.1128/mBio.02580-14 PMC4453510 25827423

[3] Garcia-Hermoso D, Janbon G, Dromer F. Epidemiological evidence for dormant Cryptococcus neoformans infection. J Clin Microbiol. 1999;37(10):3204–3209. doi: 10.1128/JCM.37.10.3204-3209.1999 10488178 PMC85528

[4] Chrétien F, Lortholary O, Kansau I, et al. Pathogenesis of cerebral *Cryptococcus neoformans* infection after fungemia. J Infect Dis. 2002;186(4):522–530. doi: 10.1086/341564 12195380

[5] Rajasingham R, Govender NP, Jordan A, et al. The global burden of HIV-associated cryptococcal infection in adults in 2020: a modelling analysis. Lancet Infect Dis. 2022;22(12):1748–1755. doi: 10.1016/S1473-3099(22)00499-6 36049486 PMC9701154

[6] Ikuta KS, Meštrović T, Naghavi M. Global incidence and mortality of severe fungal disease. Lancet Infect Dis. 2024;24(5):e268. doi: 10.1016/S1473-3099(24)00102-6 38395046

[7] Armstrong-James D, Meintjes G, Brown GD. A neglected epidemic: fungal infections in HIV/AIDS. Trends Microbiol. 2014;22(3):120–127. doi: 10.1016/j.tim.2014.01.001 24530175

[8] Baker RP, Casadevall A. Reciprocal modulation of ammonia and melanin production has implicatio ns for cryptococcal virulence. Nat Commun. 2023;14(1):849. doi: 10.1038/s41467-023-36552-7 36792633 PMC9932161

[9] Perfect JR, Dismukes WE, Dromer F, et al. Clinical practice guidelines for the management of cryptococcal disease: 2010 update by the infectious diseases society of America. Clin Infect Dis: an Off Publ Of The Infectio Us Dis Soc Of Am. 2010;50(3):291–322. doi: 10.1086/649858 PMC5826644 20047480

[10] Bongomin F, Oladele RO, Gago S, et al. A systematic review of fluconazole resistance in clinical isolates of Cryptococcus species. Mycoses. 2018;61(5):290–297. doi: 10.1111/myc.12747 29377368

[11] Mpoza E, Rhein J, Abassi M. Emerging fluconazole resistance: implications for the management of cryptococcal meningitis. Med Mycol Case Rep. 2017;19:30–32. doi: 10.1016/j.mmcr.2017.11.004 29234588 PMC5723366

[12] Loyse A, Burry J, Cohn J, et al. Leave no one behind: response to new evidence and guidelines for the management of cryptococcal meningitis in low-income and middle-income countries. Lancet Infect Dis. 2019;19(4):e143–e147. doi: 10.1016/S1473-3099(18)30493-6 30344084

[13] Pushpakom S, Iorio F, Eyers PA, et al. Drug repurposing: progress, challenges and recommendations. Nat Rev Drug Discov. 2019;18(1):41–58. doi: 10.1038/nrd.2018.168 30310233

[14] Zhang Q, Liu F, Zeng M, et al. Drug repurposing strategies in the development of potential antifungal agents. Appl Microbiol Biotechnol. 2021;105(13):5259–5279. doi: 10.1007/s00253-021-11407-7 34151414 PMC8214983

[15] Lei J, Xiao W, Zhang J, et al. Antifungal activity of vitamin D_3_ against *candida albicans in vitro* and *in vivo*. Microbiol Res. 2022;265:127200. doi: 10.1016/j.micres.2022.127200 36162148

[16] Northrop-Clewes CA, Thurnham DI. The discovery and characterization of riboflavin. Ann Of Nutr & Metab. 2012;61(3):224–230. doi: 10.1159/000343111 23183293

[17] Powers HJ. Riboflavin (vitamin B-2) and health. Am J Clin Nutr. 2003;77(6):1352–1360. doi: 10.1093/ajcn/77.6.1352 12791609

[18] Beztsinna N, Solé M, Taib N, et al. Bioengineered riboflavin in nanotechnology. Biomaterials. 2016;80:121–133. doi: 10.1016/j.biomaterials.2015.11.050 26708089

[19] TJJoA T, Chemistry F. Antioxidant effect of riboflavin in enzymic lipid peroxidation. J Agric & Food Chem. 1992;40(10):1727–1730. doi: 10.1021/jf00022a001

[20] Mal P, Dutta K, Bandyopadhyay D, et al. Azithromycin in combination with riboflavin decreases the severity of *staphylococcus aureus* infection induced septic arthritis by modulating the production of free radicals and endogenous cytokines. Inflamm Res. 2013;62(3):259–273. doi: 10.1007/s00011-012-0574-z 23229721

[21] Schrier A, Greebel G, Attia H, et al. In vitro antimicrobial efficacy of riboflavin and ultraviolet light on *staphylococcus aureus*, methicillin-resistant *staphylococcus aureus*, and Pseudomonas aeruginosa. J refractive Surg. 2009;25(9):S799–802. doi: 10.3928/1081597X-20090813-07 19772254

[22] Dey S, Bishayi B. Riboflavin along with antibiotics balances reactive oxygen species and inflammatory cytokines and controls staphylococcus aureus infection by boosting murine macrophage function and regulates inflammation. J Inflamm (lond). 2016;13(28):36. doi: 10.1186/s12950-016-0145-0 27932936 PMC5126841

[23] Suwannasom N, Kao I, Pruß A, et al. Riboflavin: the health benefits of a forgotten natural vitamin. Int J Mol Sci. 2020;21(3):950. doi: 10.3390/ijms21030950 32023913 PMC7037471

[24] Lei J, Xin C, Xiao W, et al. The promise of endogenous and exogenous riboflavin in anti-infection. Virulence. 2021;12(1):2314–2326. doi: 10.1080/21505594.2021.1963909 34490839 PMC8425684

[25] Lei J, Huang J, Xin C, et al. Riboflavin targets the cellular metabolic and ribosomal pathways of *candida albicans in vitro* and exhibits efficacy against oropharyn geal candidiasis. Microbiol Spectr. 2023;11(1):e0380122. doi: 10.1128/spectrum.03801-22 36625571 PMC9927497

[26] Montoya MC, Beattie S, Alden KM, et al. Derivatives of the antimalarial drug mefloquine are broad-spectrum antifungal molecules with activity against drug-resistant clinical isolates. Antimicrob Agents Chemother. 2020;64(3):e02331–02319. doi: 10.1128/AAC.02331-19 31907188 PMC7038245

[27] Huang J, Lei J, Ge A, et al. Antifungal effect of vitamin D_3_ against *Cryptococcus neoformans* coincides with reduced biofilm formation, compromised cell wall integrity, and increased generation of reactive oxygen species Journal of fungi. 2023; 9(7):772. doi: 10.3390/jof9070772 PMC10381739 37504760

[28] Mamoon K, Thammasit P, Iadnut A, et al. Unveiling the properties of Thai stingless bee propolis via Diminishin g cell wall-associated cryptococcal melanin and enhancing the Fungicid al activity of macrophages. Antibiotics. 2020; 9(7):420. doi: 10.3390/antibiotics9070420 PMC7400477 32709077

[29] Beattie SR, Schnicker NJ, Murante T, et al. Benzothiourea derivatives target the secretory pathway of the human fungal pathogen *cryptococcus neoformans*. ACS Infect Dis. 2020;6(3):529–539. doi: 10.1021/acsinfecdis.9b00478 32070095 PMC7664160

[30] Horianopoulos LC, Lee CWJ, Hu G, et al. Dnj1 promotes virulence in *cryptococcus neoformans* by maintaini ng robust endoplasmic reticulum homeostasis under temperature stress. Front Microbiol. 2021;12:727039. doi: 10.3389/fmicb.2021.727039 34566931 PMC8461255

[31] Vélez N, Vega-Vela N, Muñoz M, et al. Deciphering the association among pathogenicity, production and polymo rphisms of capsule/melanin in clinical isolates of *cryptococcus neoformans* var. grubii VNI. In: Journal of fungi. 2022; 8(3): 245. doi: 10.3390/jof8030245 PMC8950468 35330247

[32] Kumari P, Mishra R, Arora N, et al. Antifungal and anti-biofilm activity of essential oil active component s against *Cryptococcus neoformans* and *Cryptococcus laurentii*. Front Microbiol. 2017;8:2161. doi: 10.3389/fmicb.2017.02161 29163441 PMC5681911

[33] Kalem MC, Subbiah H, Leipheimer J, et al. Puf4 mediates Post-transcriptional regulation of cell wall biosynthesis and Caspofungin resistance in *Cryptococcus neoformans*. MBio. 2021;12(1):e03225–03220. doi: 10.1128/mBio.03225-20 33436441 PMC7844544

[34] Silva VKA, Bhattacharya S, Oliveira NK, et al. Replicative aging remodels the cell wall and is associated with increa sed intracellular trafficking in human pathogenic yeasts. MBio. 2021;13(1):e0019022. doi: 10.1128/mbio.00190-22 35164553 PMC8844920

[35] Qian W, Wang W, Zhang J, et al. Exploitation of the antifungal and antibiofilm activities of plumbagin against *cryptococcus neoformans*. Biofouling. 2022;38(6):558–574. doi: 10.1080/08927014.2022.2094260 35818738

[36] NPd S, CMd L, Lino CI, et al. Heterocycle thiazole compounds exhibit antifungal activity through Inc rease in the production of reactive oxygen species in the *Cryptococcus neoformans-Cryptococcus gattii* species complex. Antimicrob Agents Chemother. 2017;61(8):e02700–02716. doi: 10.1128/AAC.02700-16 28533240 PMC5527588

[37] Vandesompele J, De Preter K, Pattyn F, et al. Accurate normalization of real-time quantitative RT-PCR data by geometric averaging of multiple internal control genes. Genome Biol. 2002;3(7):RESEARCH0034. doi: 10.1186/gb-2002-3-7-research0034 12184808 PMC126239

[38] Hoy MJ, Park E, Lee H, et al. Structure-guided synthesis of FK506 and FK520 analogs with increased selectivity exhibit in vivo Therapeutic efficacy against cryptococcus. MBio. 2022;13(3):e0104922. doi: 10.1128/mbio.01049-22 35604094 PMC9239059

[39] Khatua S, Simal-Gandara J, Acharya K. Understanding immune-modulatory efficacy in vitro. Chem Biol Interact. 2022;352:109776. doi: 10.1016/j.cbi.2021.109776 34906553 PMC8665649

[40] Ruiz Mendoza S, Liedke SC, de La Noval C R, et al. In vitro and in vivo efficacies of dectin-1-Fc(IgG)(s) fusion proteins against invasive fungal infections. Med Mycol. 2022;60(8):myac050. doi: 10.1093/mmy/myac050 35867978

[41] Martinez LR, Casadevall A. Specific antibody can prevent fungal biofilm formation and this effect correlates with protective efficacy. Infect Immun. 2005;73(10):6350–6362. doi: 10.1128/IAI.73.10.6350-6362.2005 16177306 PMC1230912

[42] Jamieson DJ. Oxidative stress responses of the yeast *saccharomyces cerevisiae*. Yeast (Chichester. England).1998; 14(16):1511–1527. doi: 10.1002/(SICI)1097-0061(199812)14:16<1511:AID-YEA356>3.0.CO;2-S 9885153

[43] Alanio A. Dormancy in *Cryptococcus neoformans*: 60 years of accumulating evidence. J Clin Invest. 2020;130(7):3353–3360. doi: 10.1172/JCI136223 32484459 PMC7324190

[44] Stone NR, Rhodes J, Fisher MC, et al. Dynamic ploidy changes drive fluconazole resistance in human cryptococcal meningitis. J Clin Invest. 2019;129(3):999–1014. doi: 10.1172/JCI124516 30688656 PMC6391087

[45] Hamill RJ. Amphotericin B formulations: a comparative review of efficacy and toxi city. Drugs. 2013;73(9):919–934. doi: 10.1007/s40265-013-0069-4 23729001

[46] Fischer M, Bacher A Biosynthesis of vitamin B2: a unique way to assemble a xylene ring. Chembiochem. 2011;12(5):670–680. doi: 10.1002/cbic.201000681 21404408

[47] Kernien JF, Snarr BD, Sheppard DC, et al. The interface between fungal biofilms and innate immunity. Front Immunol. 2018;8:1968. doi: 10.3389/fimmu.2017.01968 29375581 PMC5767580

[48] Aslanyan L, Sanchez DA, Valdebenito S, et al. The crucial role of biofilms in *Cryptococcus neoformans* survival within macrophages and colonization of the central nervous System. Journal of fungi. 2017; 3(1):10. doi: 10.3390/jof3010010 PMC5715963 29371529

[49] Delattin N, Cammue BPA, Thevissen K. Reactive oxygen species-inducing antifungal agents and their activity against fungal biofilms. Future Med Chem. 2014;6(1):77–90. doi: 10.4155/fmc.13.189 24358949

[50] Dickey SW, Cheung GYC, Otto M. Different drugs for bad bugs: antivirulence strategies in the age of antibiotic resistance. Nat Rev Drug Discov. 2017;16(7):457–471. doi: 10.1038/nrd.2017.23 28337021 PMC11849574

[51] Kozel TR, Pfrommer GS, Guerlain AS, et al. Role of the capsule in phagocytosis of *Cryptococcus neoformans*. Rev Of Infect Dis. 1988;10(2):S436–439. doi: 10.1093/cid/10.supplement_2.s436 3055212

[52] Nurudeen TA, Ahearn DG. Regulation of melanin production by *Cryptococcus neoformans*. J Clin Microbiol. 1979;10(5):724–729. doi: 10.1128/jcm.10.5.724-729.1979 44517 PMC273255

[53] Zaragoza O. Basic principles of the virulence of *cryptococcus*. Virulence. 2019;10(1):490–501. doi: 10.1080/21505594.2019.1614383 31119976 PMC6550552

[54] Pukkila-Worley R, Gerrald QD, Kraus PR, et al. Transcriptional network of multiple capsule and melanin genes governed by the *Cryptococcus neoformans* cyclic AMP cascade. Eukaryot Cell. 2005;4(1):190–201. doi: 10.1128/EC.4.1.190-201.2005 15643074 PMC544166

[55] Cox GM, Mukherjee J, Cole GT, et al. Urease as a virulence factor in experimental cryptococcosis. Infect Immun. 2000;68(2):443–448. doi: 10.1128/IAI.68.2.443-448.2000 10639402 PMC97161

[56] Iyer KR, Revie NM, Fu C, et al. Treatment strategies for cryptococcal infection: challenges, advances and future outlook. Nat Rev Microbiol. 2021;19(7):454–466. doi: 10.1038/s41579-021-00511-0 33558691 PMC7868659

[57] Kraus PR, Fox DS, Cox GM, et al. The *Cryptococcus neoformans* MAP kinase Mpk1 regulates cell integrity in response to antifungal drugs and loss of calcineurin function. Mol Microbiol. 2003;48(5):1377–1387. doi: 10.1046/j.1365-2958.2003.03508.x 12787363 PMC1635492

[58] Esher SK, Ost KS, Kohlbrenner MA, et al. Defects in intracellular trafficking of fungal cell wall synthases lead to aberrant host immune recognition. PLoS Pathog. 2018;14(6):e1007126. doi: 10.1371/journal.ppat.1007126 29864141 PMC6002136

[59] Perrone GG, Tan S-X, Dawes IW. Reactive oxygen species and yeast apoptosis. Biochim Biophys Acta. 2008;1783(7):1354–1368. doi: 10.1016/j.bbamcr.2008.01.023 18298957

[60] Mukaremera L, Nielsen K. Adaptive immunity to Cryptococcus neoformans infections. Journal of fungi. 2017 ; 3(4): 64. doi: 10.3390/jof3040064 PMC5753166 29333430

[61] Verdrengh M, Tarkowski A. Riboflavin in innate and acquired immune responses. Inflamm Res. 2005;54(9):390–393. doi: 10.1007/s00011-005-1372-7 16273338

[62] Santiago-Tirado FH, Onken MD, Cooper JA, et al. Trojan horse transit contributes to blood-brain barrier crossing of a eukaryotic pathogen. MBio. 2017;8(1):e02183–16. doi: 10.1128/mBio.02183-16 28143979 PMC5285505

[63] McQuiston TJ, Williamson PR. Paradoxical roles of alveolar macrophages in the host response to *Cryptococcus neoformans*. J Infect And Chemother: off J Jpn Soc Of Chemother. 2012;18(1):1–9. doi: 10.1007/s10156-011-0306-2 PMC4035814 22045161

[64] Rendra E, Riabov V, Mossel DM, et al. Reactive oxygen species (ROS) in macrophage activation and function in diabetes. Immunobiology. 2019;224(2):242–253. doi: 10.1016/j.imbio.2018.11.010 30739804

[65] leon-Rodriguez Cm D, Rossi DCP, Fu MS, et al. The outcome of the *Cryptococcus neoformans* macrophage interaction depends on phagolysosomal membrane integrity. J Immunol (baltim, Md:1950). 2018;201(2):583–603. doi: 10.4049/jimmunol.1700958 PMC6245949 29858266

