# Supplementary figures and images for: Riboflavin inhibits growth and reduces virulence of Cryptococcus neoformans in vitro by membrane disruption and excessive accumulation of reactive oxygen species and exhibits efficacy against pulmonary cryptococcosis and meningitis
Source: Virulence. 2025 Aug 3;16(1):2543064. doi: 10.1080/21505594.2025.2543064 (PMC12333042; doi:10.1080/21505594.2025.2543064)

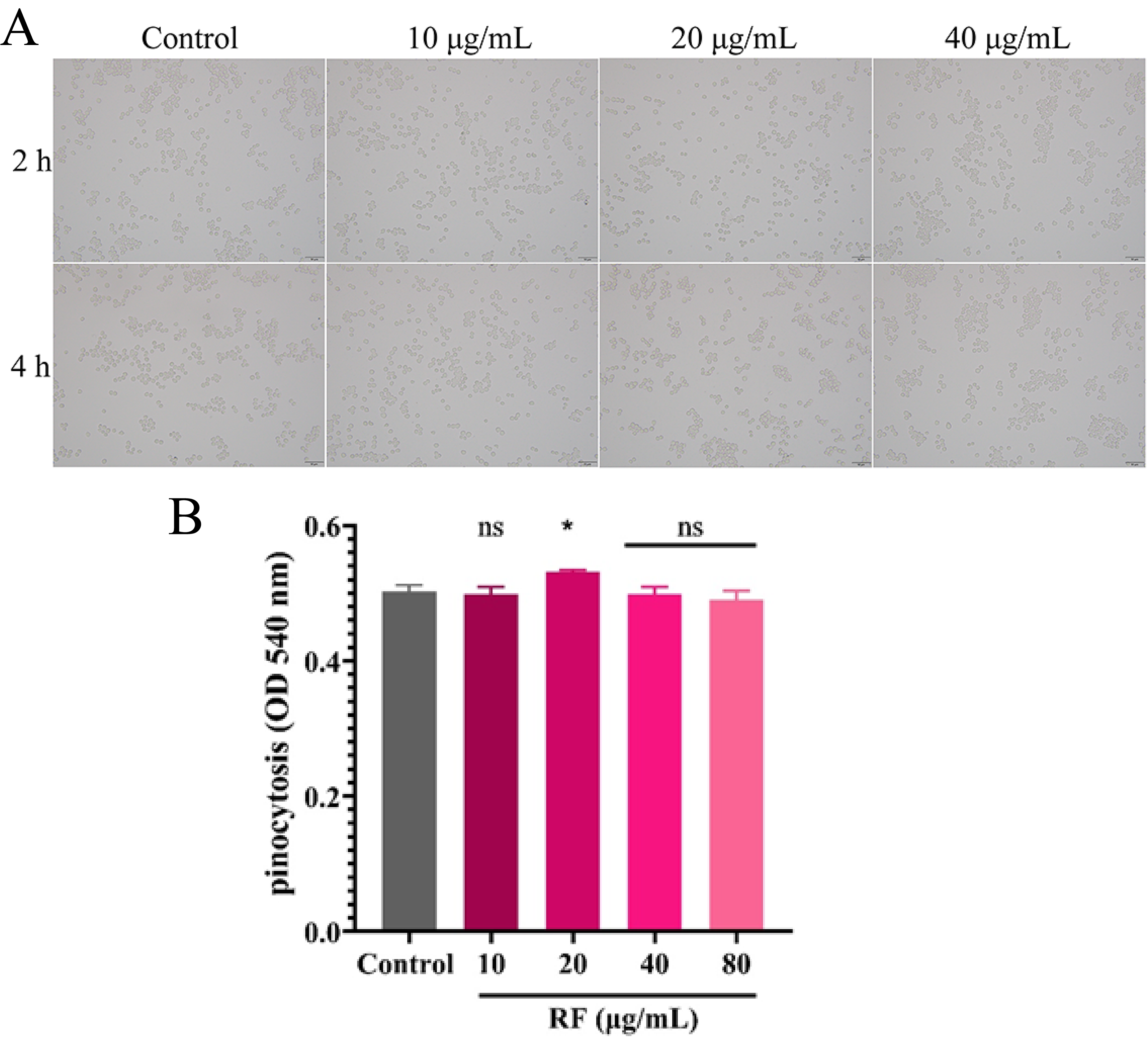

Supplement: FigS9.tif [file KVIR_A_2543064_SM3927.tif]

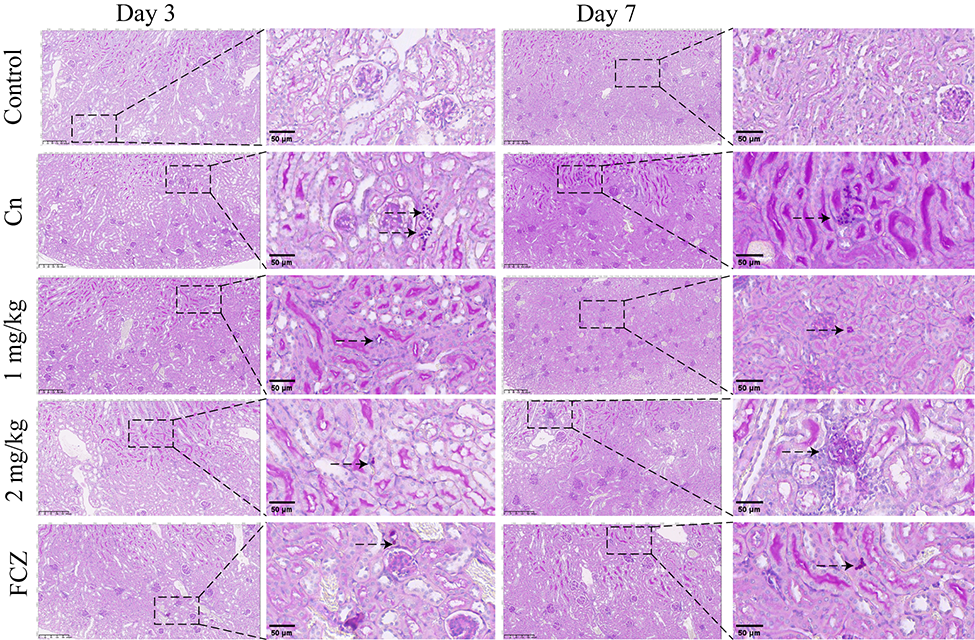

Supplement: FigS7.tif [file KVIR_A_2543064_SM3926.tif]

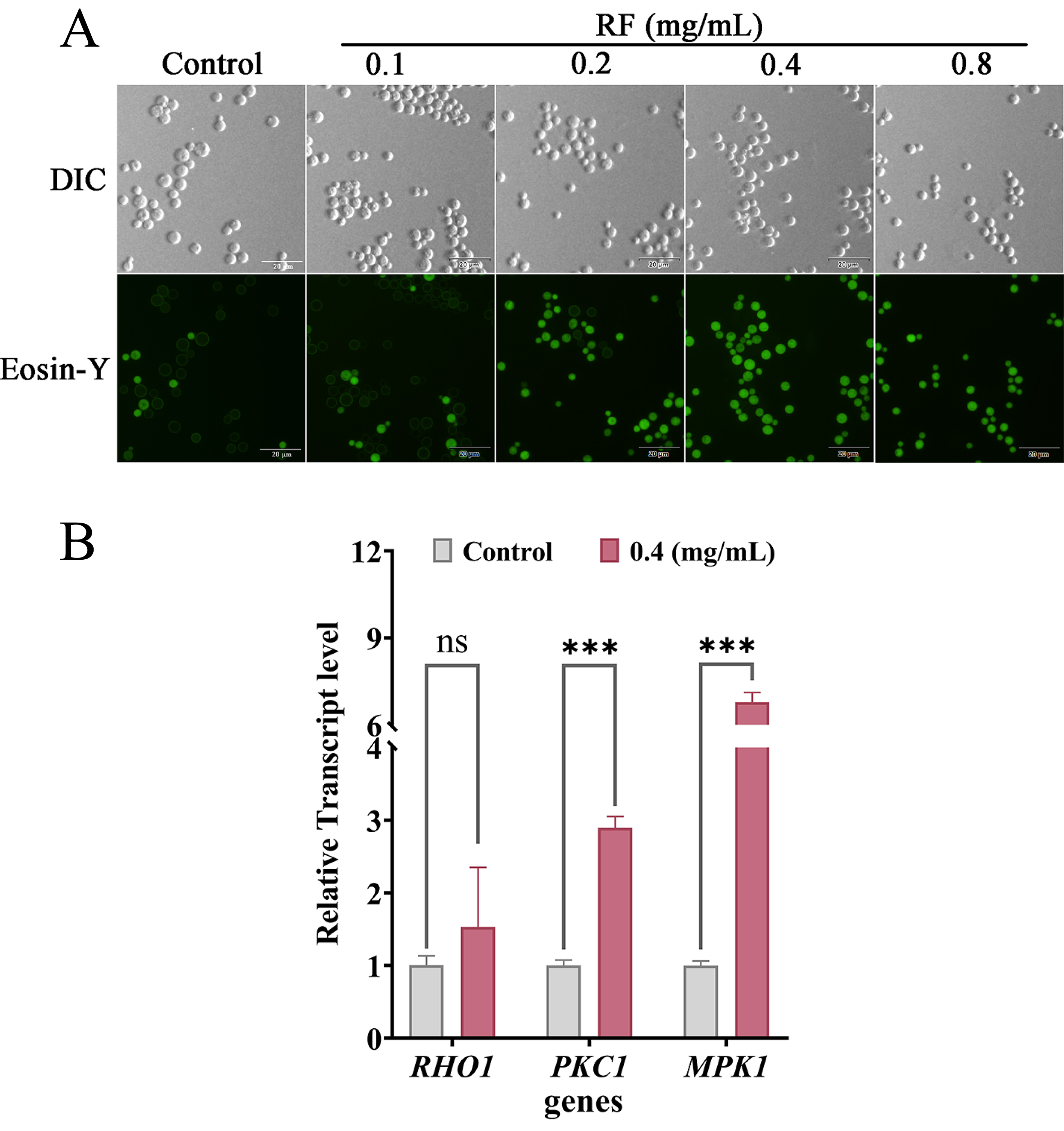

Supplement: FigS2.tif [file KVIR_A_2543064_SM3925.tif]

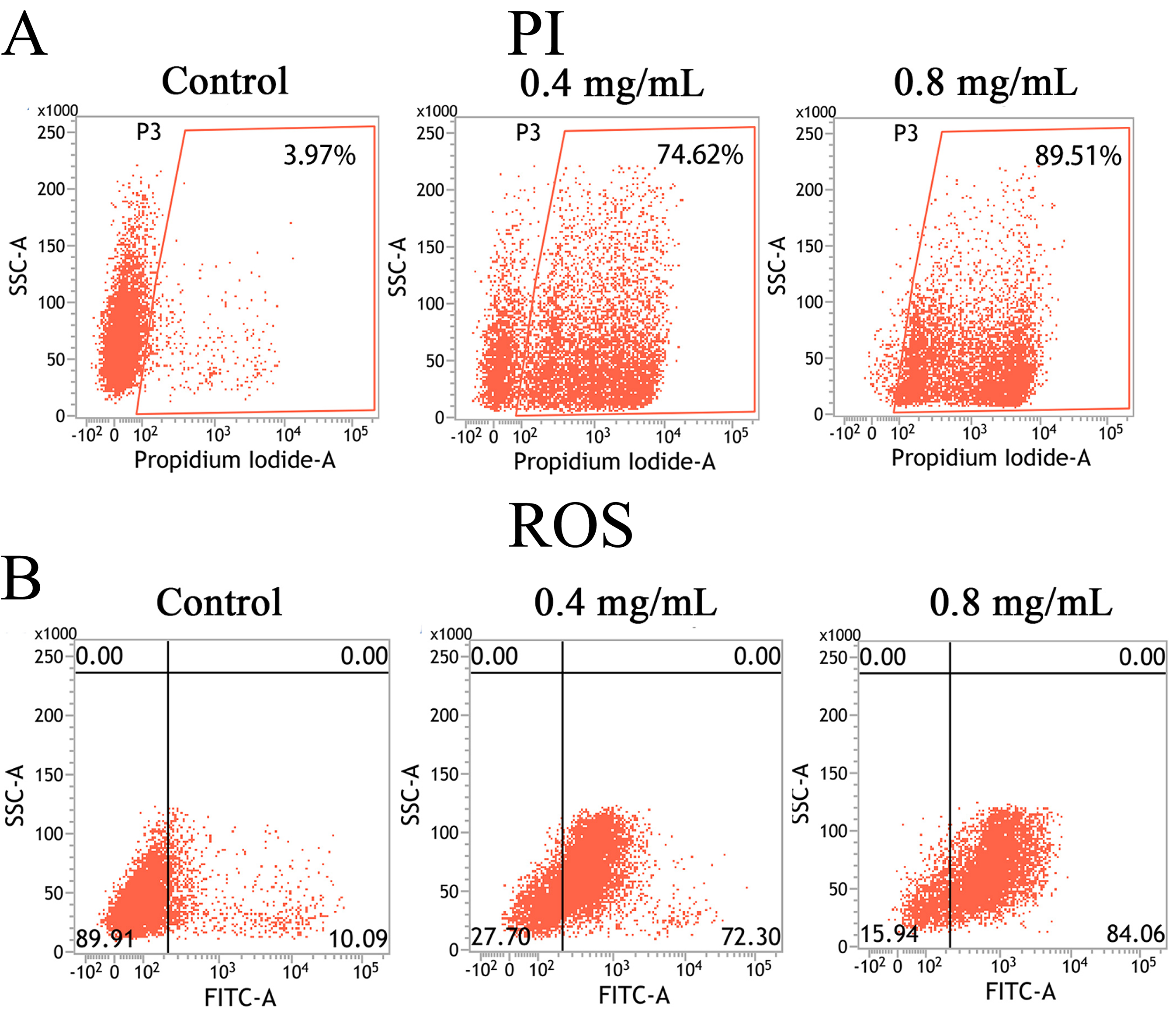

Supplement: FigS3.tif [file KVIR_A_2543064_SM3923.tif]

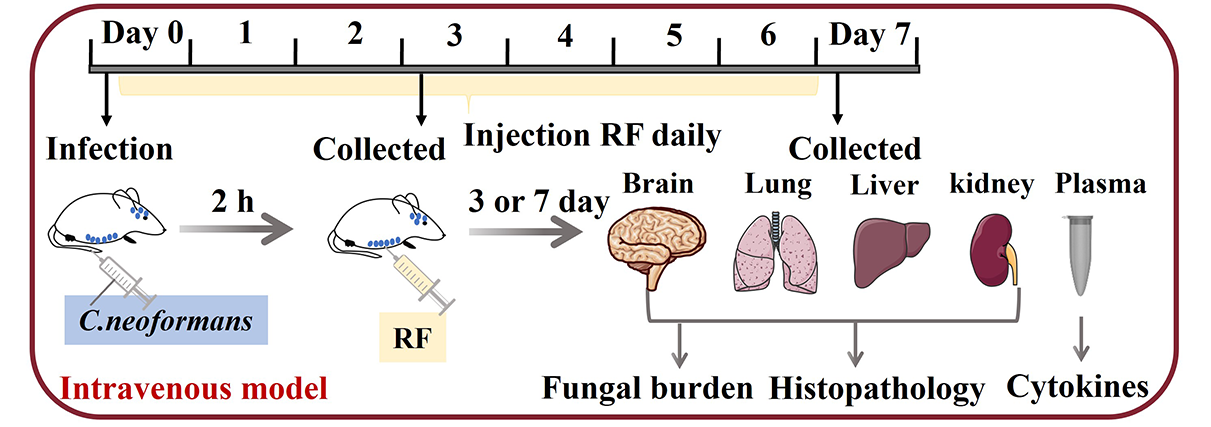

Supplement: FigS4.tif [file KVIR_A_2543064_SM3922.tif]

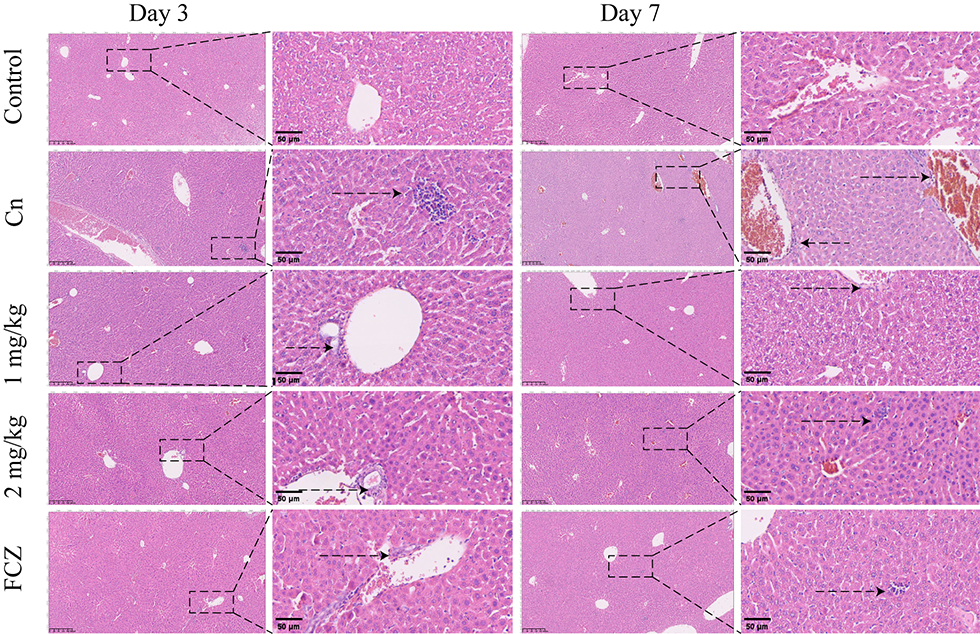

Supplement: FigS8.tif [file KVIR_A_2543064_SM3921.tif]

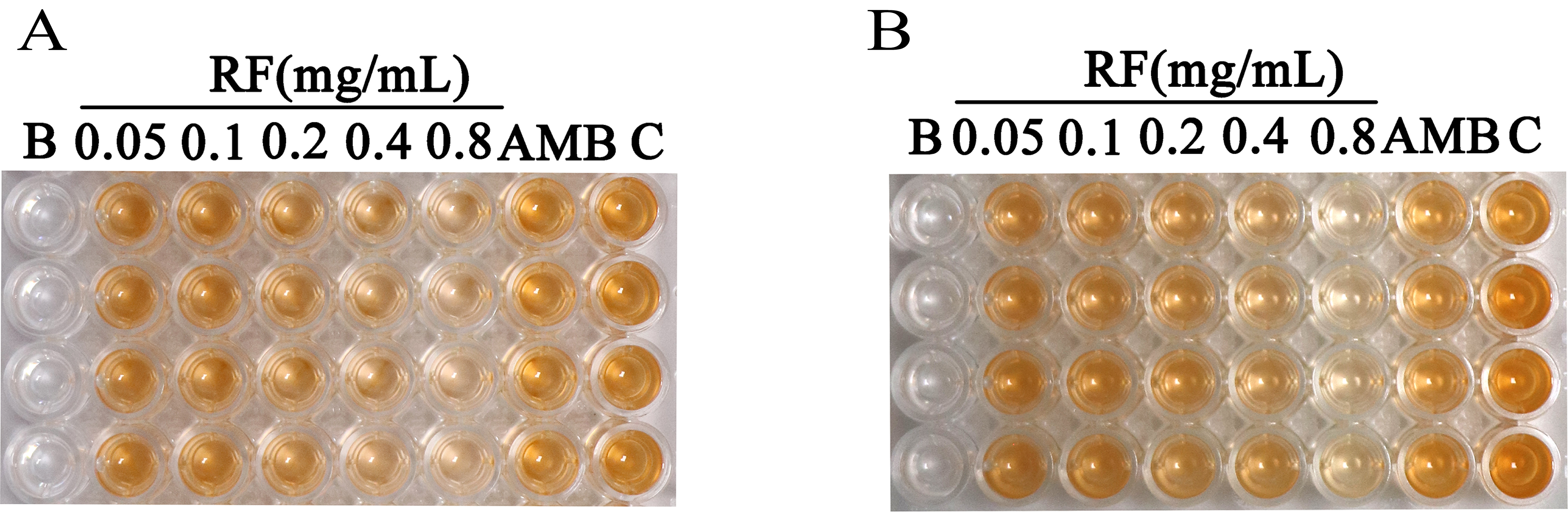

Supplement: Fig S1.tif [file KVIR_A_2543064_SM3920.tif]

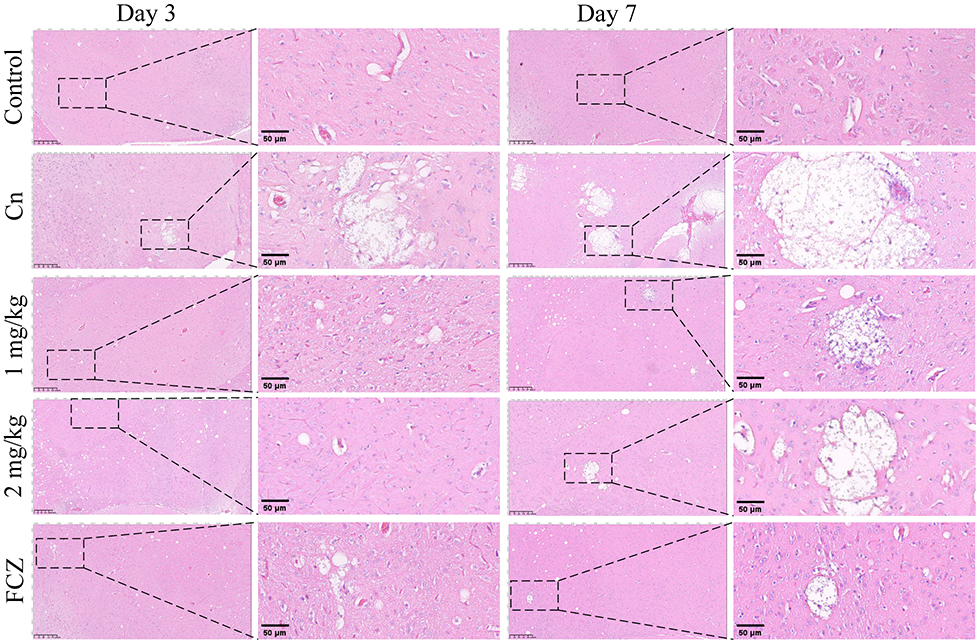

Supplement: FigS6.tif [file KVIR_A_2543064_SM3919.tif]

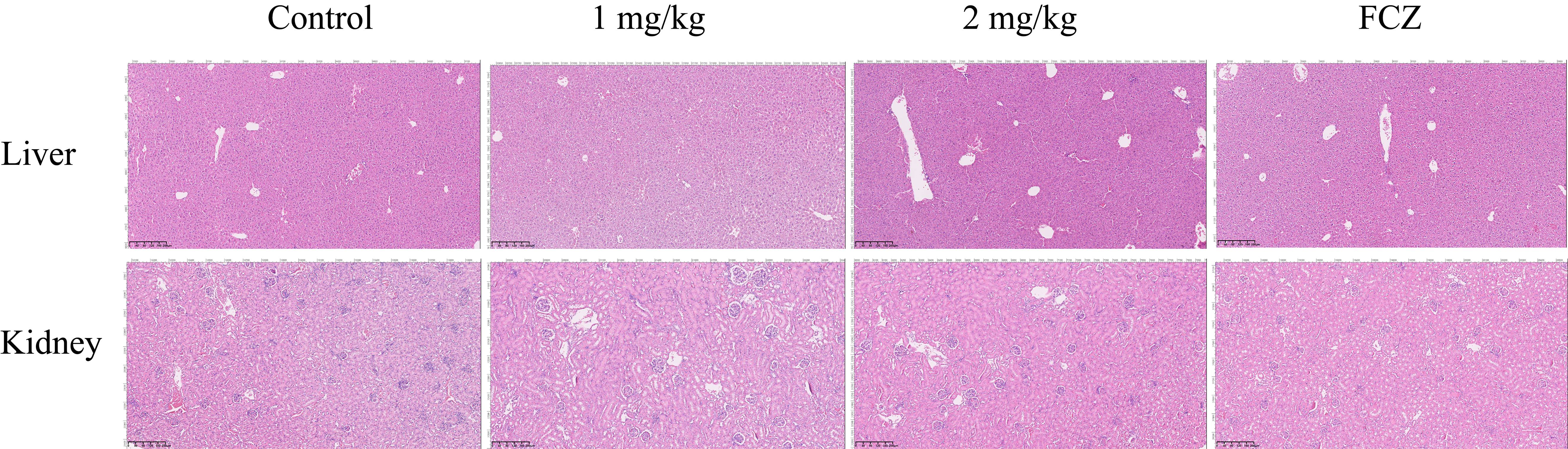

Supplement: Fig S10.jpg [file KVIR_A_2543064_SM3918.jpg]

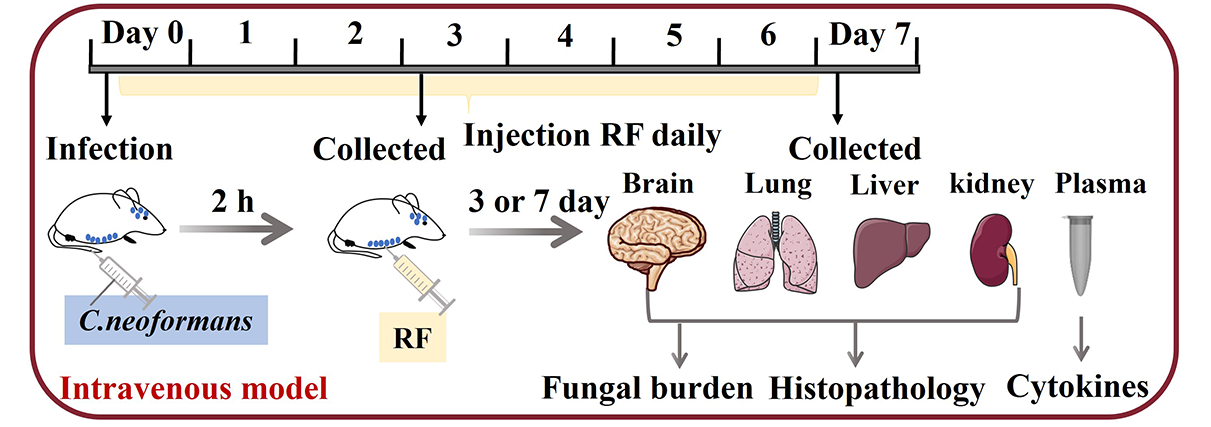

Supplement: FigS5.tif [file KVIR_A_2543064_SM3917.tif]
