# Supplementary material for: Riboflavin inhibits growth and reduces virulence of Cryptococcus neoformans in vitro by membrane disruption and excessive accumulation of reactive oxygen species and exhibits efficacy against pulmonary cryptococcosis and meningitis
Source: Virulence. 2025 Aug 3;16(1):2543064. doi: 10.1080/21505594.2025.2543064 (PMC12333042; doi:10.1080/21505594.2025.2543064)
Supplement: Supplementary_Material (1).docx [file KVIR_A_2543064_SM3924.docx]

Table S1. Nucleotide sequences of primers used for RT-qPCR analysis.

| **Gene** | **Primer Sequence (5’-3’)** |
| --- | --- |
| *GPD1 F* | AGTATGACTCCACACATGGTCG |
| *GPD1 R* | AGACAAACATCGGAGCATCAGC |
| *CHS3 F* | ACCCAGGTCTGGCATTCC |
| *CHS3 R* | AGGATCAACATTGGAAGC |
| *CHS4 F* | CGGTCTTCAGGCATTGATTT |
| *CHS4 R* | TTCGGAGTGAAGTGATGCTG |
| *CHS5 F* | GCTTGGATGATCTTCTATATCTG |
| *CHS5 R* | TACCTTCATCATGGATGACA |
| *CHS6 F* | TTGACCCTTGGCACATCT |
| *CHS6 R* | GTTGGCATAAGTATCCTT |
| *CDA1 F* | TCGAGCTATTGCTGCTCAGA |
| *CDA1 R* | GCTGGTAGATGTCGTGCTCA |
| *CDA2 F* | GTAACGAGGTCGTCTTTG |
| *CDA2 R* | TGTAGTTGGTGAGCTCGT |
| *CDA3 F* | ATGTGGCCGATGCTTTTAAC |
| *CDA3 R* | GAAGTGAGAAGGCCTGTTGG |
| *CDA4 F* | CGAACTCTCCAAGGTCGAAG |
| *CDA4 R* | GCGTCTCCAGTGTCATCAGA |
| *AGS1 F* | ATCCTTATCCGTTATTCC |
| *AGS1 R* | AGCTGTTCCTCTAGCGAGC |
| *FKS1 F* | TGGACTGGTGTTTGGTTCAA |
| *FKS1 R* | GTACAAAAGACCGTACTTG |
| *SKN1 F* | CTGGACAATGTATGCGGATG |
| *SKN1 R* | TCCGCAGTGGGATAATCTTC |
| *KRE6 F* | GTCTCGGAAGGCGACTCAT |
| *KRE6 R* | TCAACTCATTCTTTGGGAAGG |
| *LAC1 F* | TACAACTTTCCCCGACCTC |
| *LAC1 R* | GATGGAGAAGGTGAGCGTC |
| *LAC2 F* | TGTATGGCGCAAGGGGTTACT |
| *LAC2 R*  *CAP59F*  *CAP59R*  *URE1F*  *URE1R*  *RHO1F*  *RHO1R*  *PKC1F*  *PKC1R*  *MPK1F*  *MPK1R* | AGAACACGACTCTCCAAAGC  AACCGAACGAAGAAACCTC  ACCCCAGCACCACACATACTC  TCGTATCGGTGAAGTCGTCACT  GGACCACGGAATTGCTTCAT  CGACGATGGCAAGACTATCC  CCTAAAGCACCACACAACCCT  GAGGGAGACGAGGAAAAAATC  GTTTGGTGACAAGTGAGGGC  GTATCCTAACGCATCTCCCTTG  GGAAACTTCTGACCTCTTCGAG |


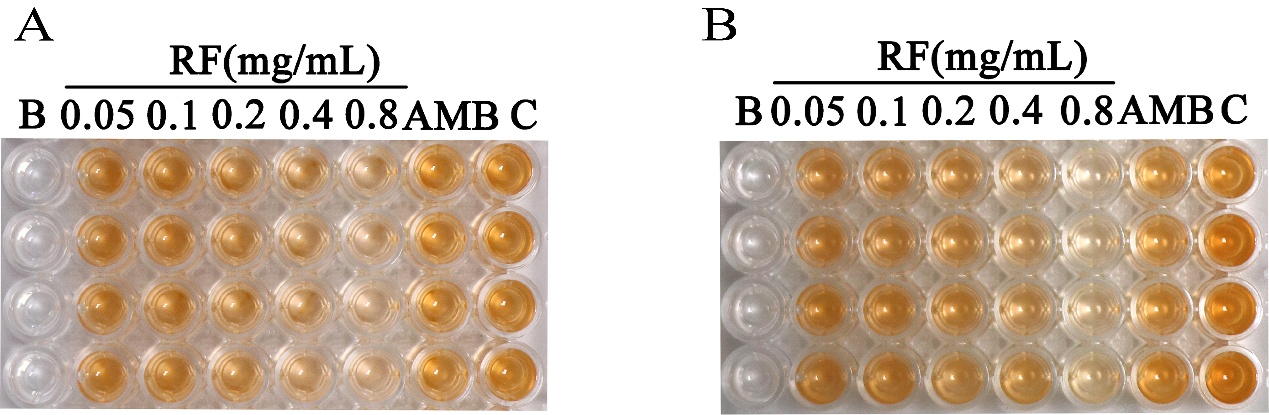


Figure S1. Effects of RF on biofilm formation and dispersion of *C. neoformans*. (A) Effect of RF on biofilm formation (48 h). (B) Effect of RF on the viability of dispersed cells from mature biofilms (precultured 48 h).


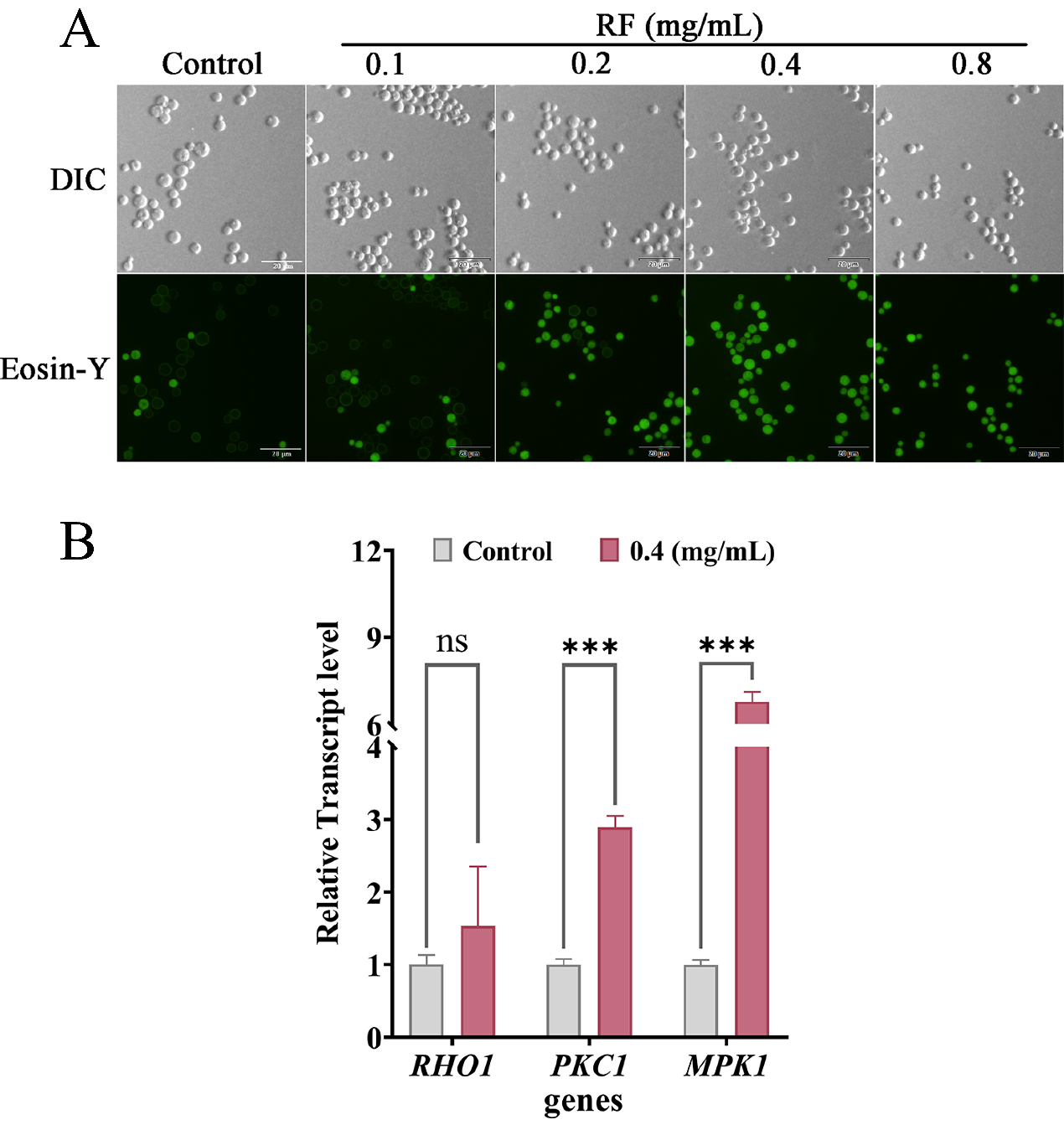


Figure S2. RF induced damage to the cell wall of *C. neoformans*. (A) Eosin Y staining of chitosan in the cell wall photographed under an inverted fluorescence microscope. Bar: 20 μm. (B) Cell wall integrity pathway as determined by RT-qPCR analysis. Data were analyzed by one-way ANOVA and the *t*-test (ns, *p* > 0.05; ^***^*p* < 0.001).


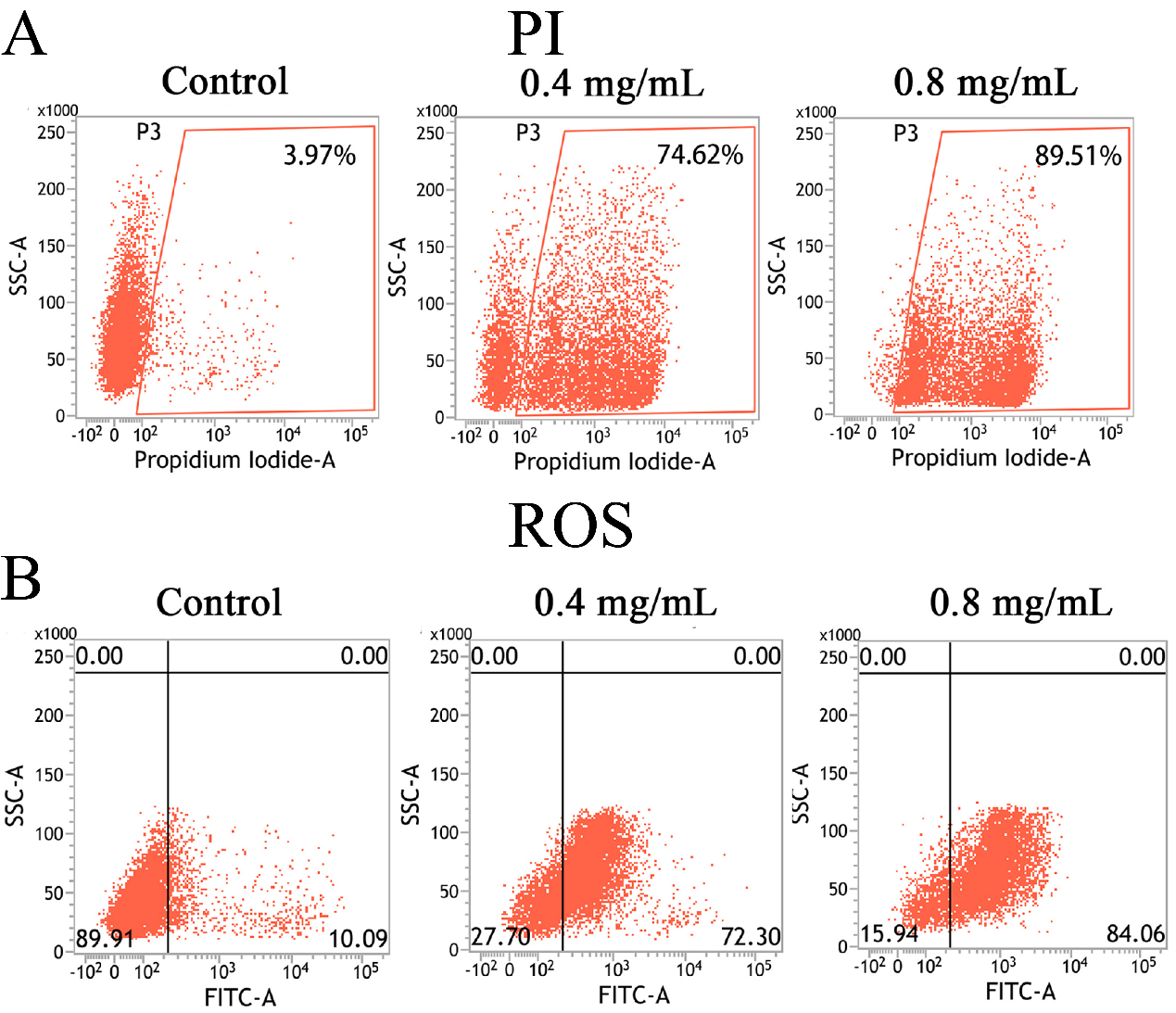


Figure S3. Effect of RF on cell membrane integrity and intracellular accumulation of ROS. Fungus stained with PI (A) and DCFH-DA (B) were assessed by a flow cytometry.


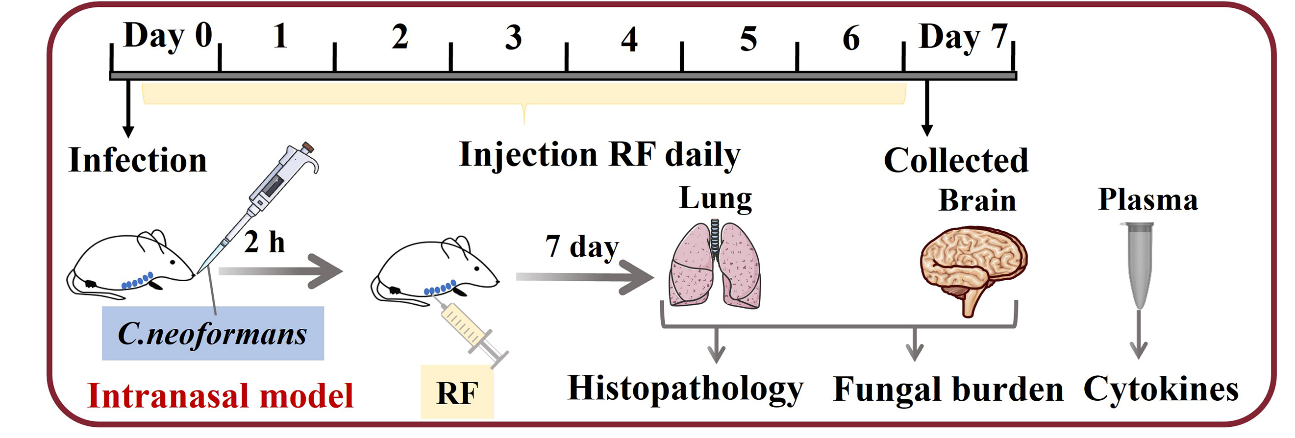


Figure S4. Mouse model of intranasal infection. Fungal infection dose was 10^6^ cells per mouse. 5 mice were per group.


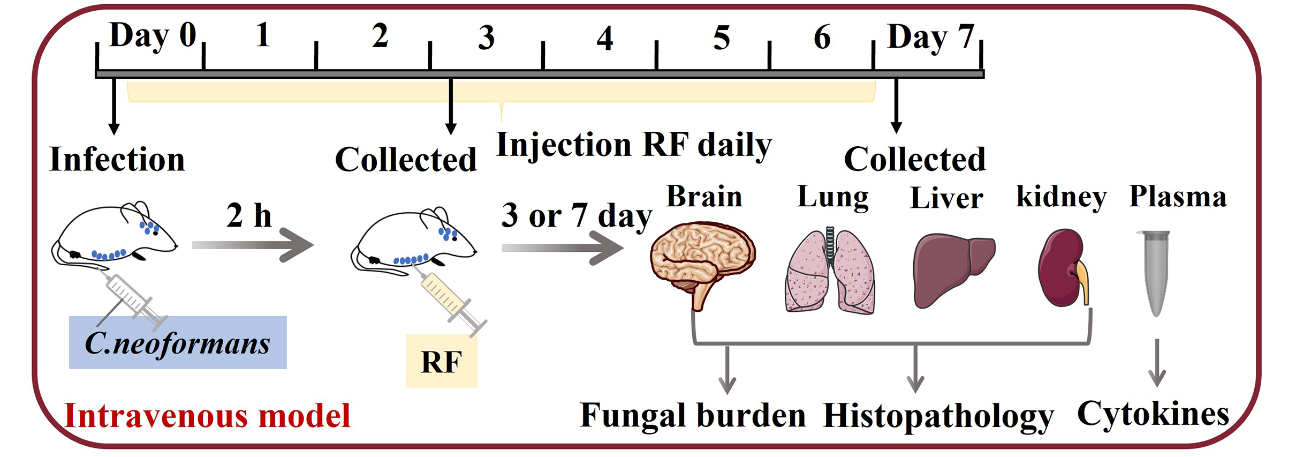


Figure S5. Mouse model of intravenous infection. Fungal infection dose was 10^5^ cells per mouse. 5 mice were per group.


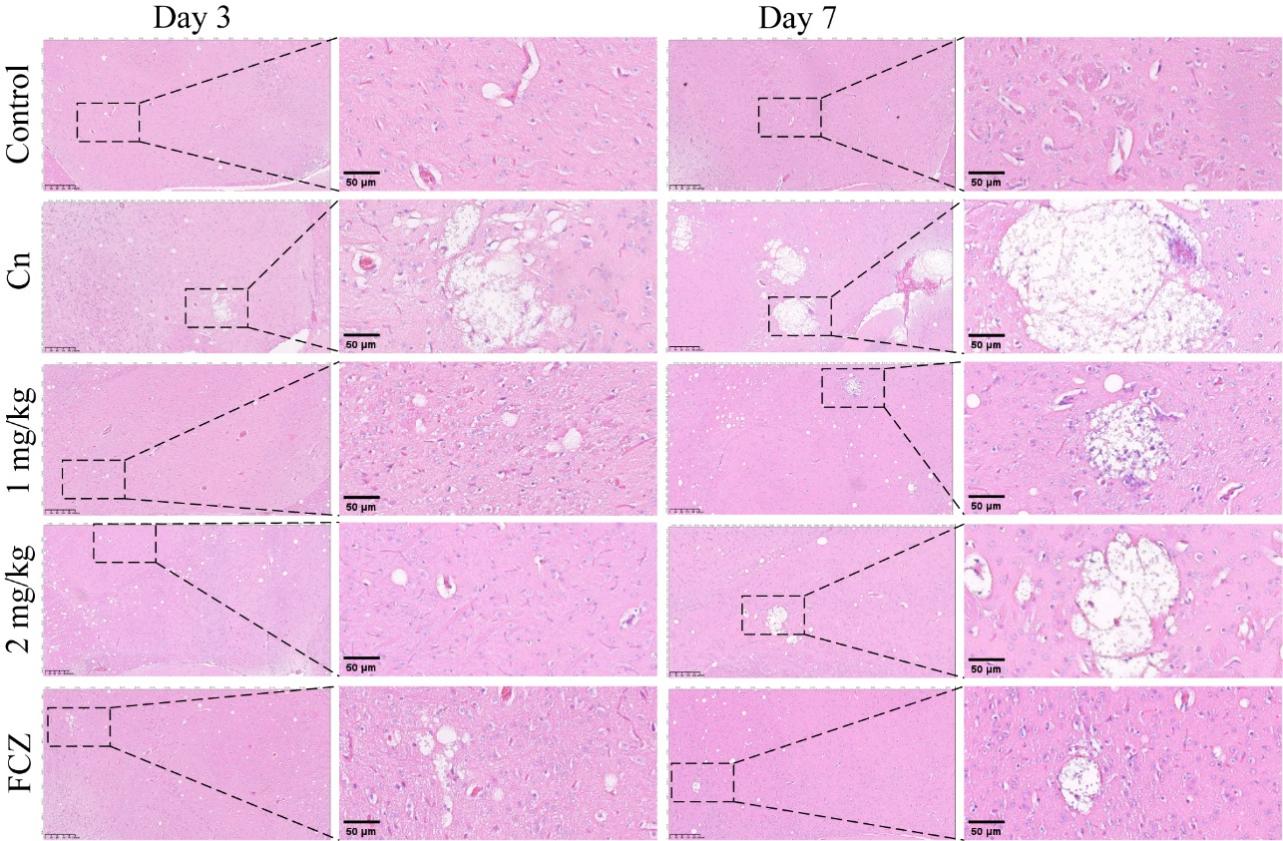


Figure S6. Efficacy of RF against intravenous infection *in vivo*. Pathological analysis of brain tissue (stained with H&E). Black boxes, site of lesion. Bar: 200 μm and 50 μm.


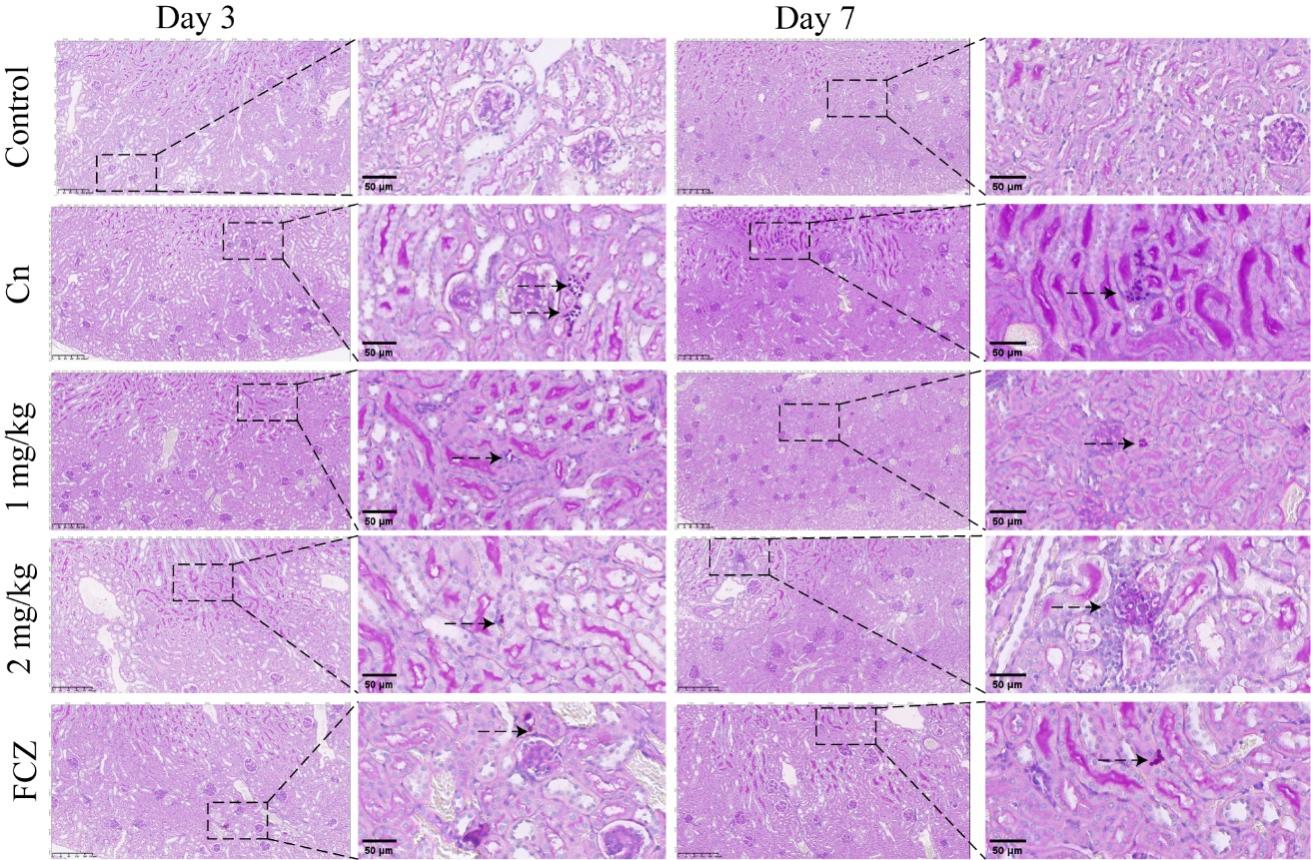


Figure S7. Efficacy of RF against intravenous infection *in vivo*. PAS staining of mouse kidney tissue. Black arrows, *C. neoformans*. Bar: 200 μm and 50 μm.


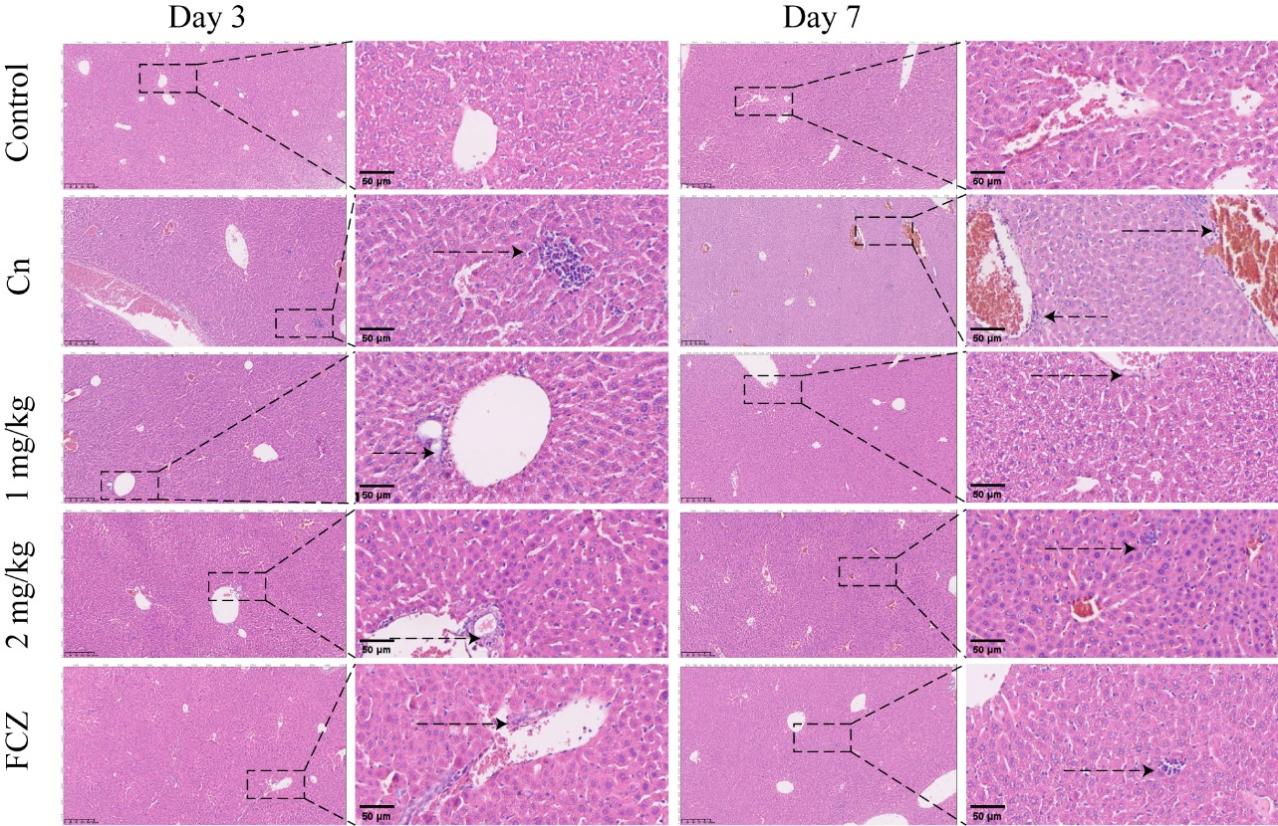


Figure S8. Efficacy of RF against intravenous infection *in vivo*. H&E staining of mouse liver tissue. Bar: 200 μm and 50 μm. Black arrows, inflammatory cells.


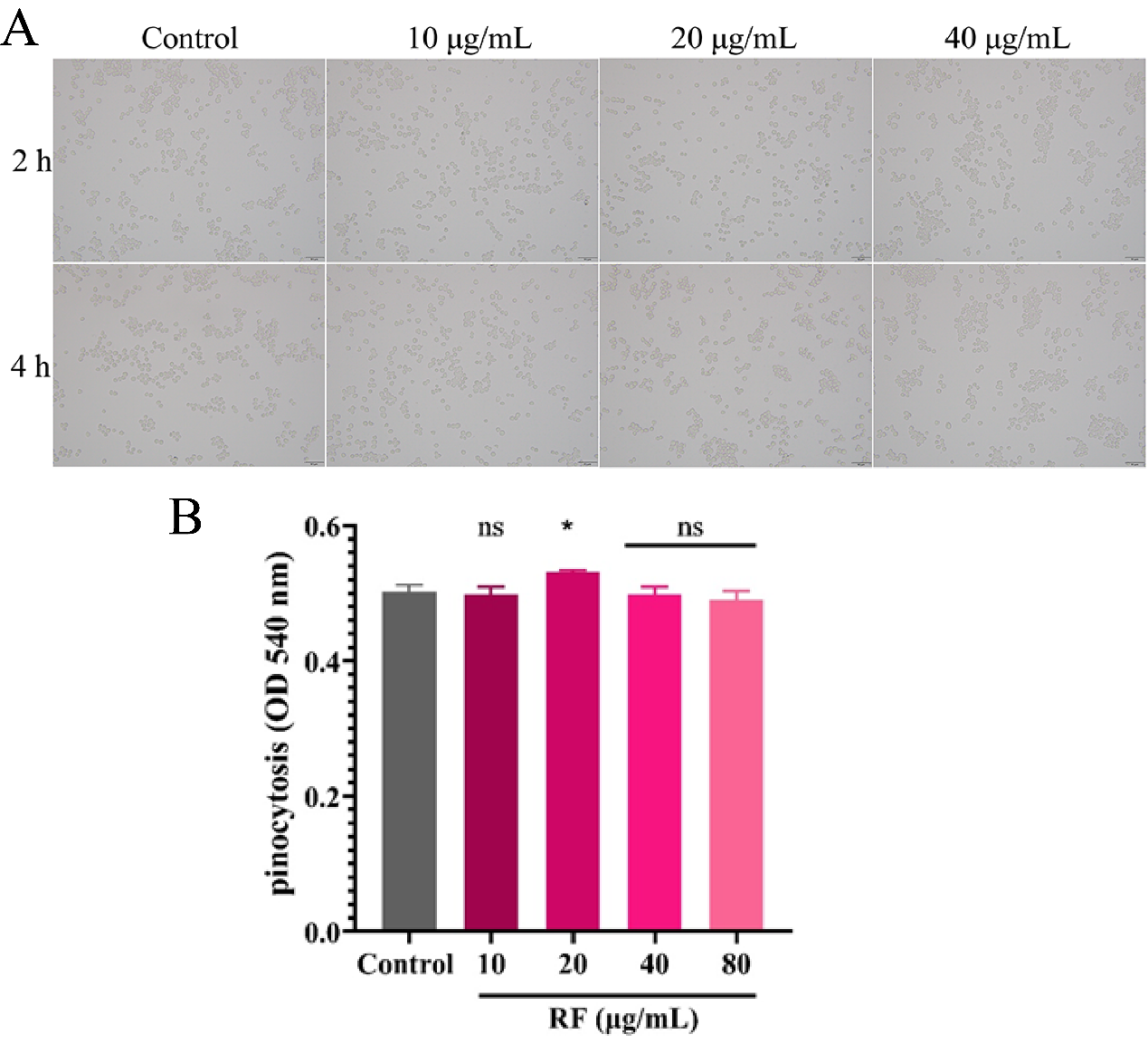


Figure S9. Effects of RF on cell morphology of MH-S. (A) RF were added to MH-S cells for 2 h and 4 h, and the cell morphology was directly observed under an inverted microscope. Scale: 50 μm. (B) Effect of RF on pinocytosis activity of MH-S macrophages. RF was incubated with MH-S cells for 2 h and the pinocytosis activity of macrophages was determined by neutral red assay. Data were analyzed by one-way ANOVA (ns, *P* > 0.05; ^*^, *P* < 0.05).


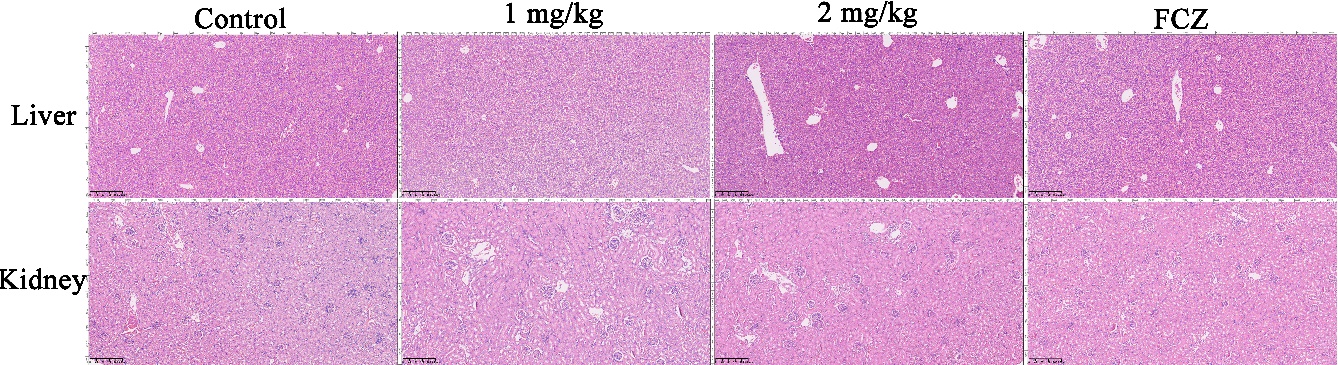


Figure S10. Safety evaluation of RF *in vivo*. After 7 days of RF injection in uninfected mice, hematoxylin eosin staining was used to assess liver and kidney tissue damage. FCZ, Fluconazole. Bar: 200 μm.
